# Mesoderm-specific *Stat3* deletion affects expression of *Sox9* yielding Sox9-dependent phenotypes

**DOI:** 10.1371/journal.pgen.1006610

**Published:** 2017-02-06

**Authors:** Michael D. Hall, Caroline A. Murray, Michael J. Valdez, Alan O. Perantoni

**Affiliations:** The Cancer and Developmental Biology Laboratory, National Cancer Institute-Frederick, Frederick, Maryland, United States of America; Max Planck Institute for Molecular Genetics, GERMANY

## Abstract

To date, mutations within the coding region and translocations around the *SOX9* gene both constitute the majority of genetic lesions underpinning human campomelic dysplasia (CD). While pathological coding-region mutations typically result in a non-functional SOX9 protein, little is known about what mechanism(s) controls normal *SOX9* expression, and subsequently, which signaling pathways may be interrupted by alterations occurring around the *SOX9* gene. Here, we report the identification of Stat3 as a key modulator of *Sox9* expression in nascent cartilage and developing chondrocytes. *Stat3* expression is predominant in tissues of mesodermal origin, and its conditional ablation using mesoderm-specific *TCre*, *in vivo*, causes dwarfism and skeletal defects characteristic of CD. Specifically, *Stat3* loss results in the expansion of growth plate hypertrophic chondrocytes and deregulation of normal endochondral ossification in all bones examined. Conditional deletion of *Stat3* with a *Sox9Cre* driver produces palate and tracheal irregularities similar to those described in *Sox9*^*+/-*^ mice. Furthermore, mesodermal deletion of *Stat3* causes global embryonic down regulation of *Sox9* expression and function *in vivo*. Mechanistic experiments *ex vivo* suggest Stat3 can directly activate the expression of *Sox9* by binding to its proximal promoter following activation. These findings illuminate a novel role for Stat3 in chondrocytes during skeletal development through modulation of a critical factor, *Sox9*. Importantly, they further provide the first evidence for the modulation of a gene product other than *Sox9* itself which is capable of modeling pathological aspects of CD and underscore a potentially valuable therapeutic target for patients with the disorder.

## Author summary

Campomelic (Greek: “bent limb”) dysplasia is an often-lethal, autosomal-dominant genetic disorder. Typical clinical features include angular long bones, hypoplastic scapulae, cleft palate, clubbed feet, labored breathing and ambiguous external genitalia. To date, the only gene implicated in this disease is *SOX9*, a critical factor in chondrocyte development. Deleterious mutations within the coding region of *SOX9* account for a majority of cases; however, chromosomal breakages or translocations mark a subset of cases, presumably by altering expression of *SOX9*. We have found that *Stat3* loss-of-function mutant mice exhibit features consistent with campomelic dysplasia including dwarfism, bent limbs, cleft palate, laryngotracheomalacia and abnormal growth plate hypertrophic chondrocytes. Importantly, we also demonstrate that ablation of *Stat3* from chondroprogenitors reduces the functional level of Sox9 *in vivo*. Finally, we show that Stat3 may directly regulate *Sox9* expression by physically interacting with the promoter in response to stimulation. Taken in total, our findings demonstrate that non-coding region mutations in *SOX9*, which modulate the accessibility or functionality of Stat-binding elements, may result in decreased physiological levels of SOX9. Our model suggests for the first time, the modulation of a gene other than *Sox9* that is capable of recapitulating a large subset of pathologies associated with campomelic dysplasia, which may be exploited for future therapeutic intervention.

## Introduction

Campomelic dysplasia (CD) is a rare, autosomal-dominant and often lethal genetic disorder, whose Greek etymology captures the characteristic “bent limbs” observed in affected pediatric patients [1]. Clinical features of CD can include laryngotracheomalacia, Pierre Robin sequence with cleft palate, loss of one pair of ribs, scoliosis/kyphosis, clubbed feet, ambiguous external genitalia and a high infant mortality rate [2]. While the radiographic appearance of bent bones is manifest in several skeletal disorders, to date, the genetic lesions associated with nearly 95% of CD patients affect either the expression or function of the SRY-box 9 (*SOX9*) transcription factor [2–5]. SOX9 is a critical factor in the regulation of chondrogenesis and subsequently endochondral ossification, the process by which the majority of the bones in the skeleton are mineralized in response to a cartilage template precursor (reviewed in [6]). Mice genetically engineered with a deletion of a single *Sox9* allele exhibit nearly all clinical features of CD, thus reinforcing the importance of this gene in normal development and in the pathology of CD [7]. Importantly, CD can arise in a subset of cases absent a deleterious mutation within the open reading frame (ORF), where chromosomal rearrangements occur in breakpoints 50Kb to 1Mb upstream of *SOX9* [3, 8–10]. Multiple *in vivo* analyses of the 1Mb upstream region demonstrate that alterations along a continuum distal to the *SOX9* ORF modulates its normal expression, suggesting that CD can arise as a function of SOX9 gene dosage [11–14]. Despite our understanding of the genetics associated with CD, we know strikingly little about normal signaling events and their integration with the regulatory region of *SOX9* to achieve proper skeletogenesis. Interestingly, genetic lesions that occur in the presumptive regulatory regions or within the coding region and result in diminished but not abolished transactivation, seem to correlate with milder forms of CD, often allowing for survival through the neonatal period [5, 8]. These clinical data further highlight the need to elucidate normal modulators of *SOX9* expression, as their characterization could introduce avenues for therapy.

Signal Transducer and Activator of Transcription (STAT) proteins are latent cytoplasmic transcription factors activated in response to myriad cytokine and growth factor signal events [15]. The most well studied members of this family include Stat1, Stat3, and Stats 5a and 5b. While largely investigated in the context of inflammation and immune biology, our lab has become interested in their roles during embryonic development, specifically with regard to the mesodermal germ layer and kidney morphogenesis. With the exception of *Stat3*, knockout mouse models targeting the individual *Stat* genes produce viable adult mice. For *Stats 1*, *2*, *4* and *6*, the characterized defects are almost exclusively found in suppression of the animal’s immune response [16–22]. In the case of *Stat5*, individual gene targeting results in defects in either mammary gland lactogenesis (*Stat5a*) or as a sexually dimorphic response to growth hormone (*Stat5b*) [23, 24]. Due to the high degree of homology between *Stats 5a* and *b* (96%), a dual knockout mouse was developed and characterized only to reveal a modest change in adult body weight and female infertility [25]. As alluded to earlier, a significant challenge to directly studying Stat3 in development is the failure of *Stat3*^*-/-*^ embryos to undergo gastrulation, highlighting the importance of Stat3 in germ layer formation and embryonic viability [26]. Furthermore, when a co-receptor for Stat3 activation, *gp130*, is knocked out *in vivo* the resulting mice also suffer embryonic lethality due to multiple defects including heart development and hematopoiesis beginning around 12.5 days post-conception [27].

To date there has been no broad loss-of-function developmental study of *Stat3*, directly, in post-gastrulation mouse models, despite evidence supporting a critical role for the pathway after germ layer specification. Here we report that conditional deletion of *Stat3* from newly formed mesoderm leads to mutant mice that survive to birth but die early in the post-natal period and exhibit severe skeletal dysmorphologies reminiscent of *Sox9* haploinsufficiency. By targeting *Stat3* prior to the formation of mesenchymal condensations, which give rise to the skeletal elements, we demonstrate the importance of Stat3 in regulating the hypertrophic chondrocyte zone. Additionally, we show the importance of Stat3 in palate closure and the maturation of tracheal cartilage, features consistent with CD pathology. Stat3 is required for normal levels of functional Sox9 *in vivo*, and further, directly regulates the expression of *Sox9* in cultured cell models. This study provides the first comprehensive evidence for a novel modulator of *SOX9* inducing haploinsufficiency-associated or CD-like pathology *in vivo*, and may begin to explain the etiology of acampomelic or mild campomelic CD where mutations outside of the coding region of *SOX9* are at the root of the disease.

## Results

### *Stat3* mRNA is enriched in mesodermal tissues

**Fig 1:**
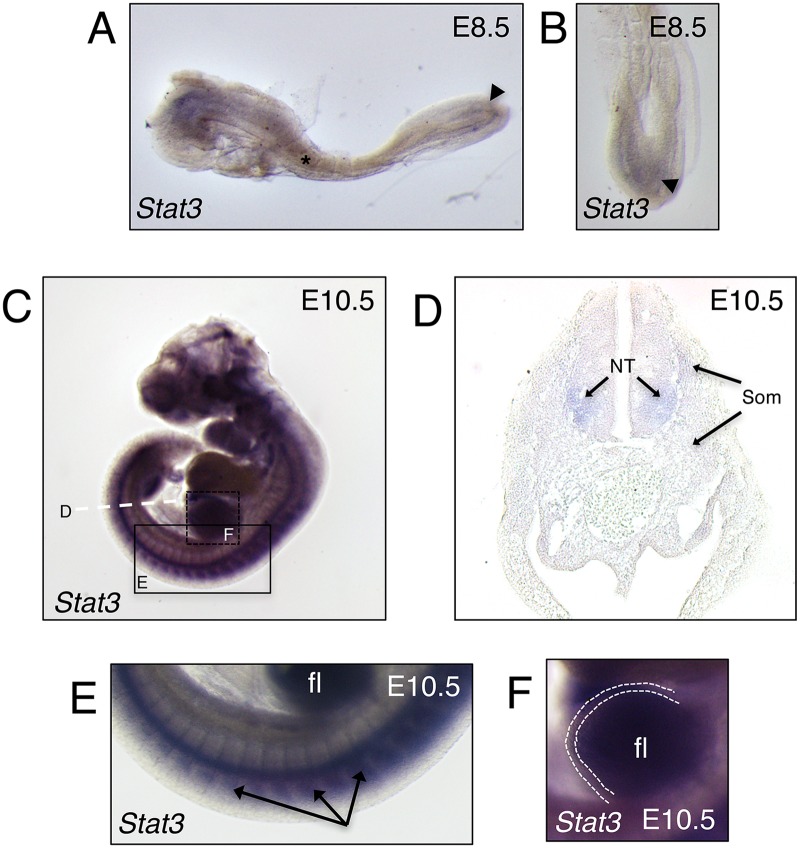
Characterization of mid-gestation *Stat3* expression in mouse. (**A**) Lateral view of wild-type mouse embryo at E8.5 analyzed for the expression of Stat3 by whole mount in situ hybridization (WISH). Asterisk indicates somite and arrowhead indicates presomitic mesoderm. (**B**) Dorsal view of posterior region from A, arrowhead indicates presomitic mesoderm. (**C**) Lateral view of wild-type mouse embryo at E10.5 analyzed for the expression of Stat3 by WISH. (**D**) Transverse section denoted in C by a dashed white line, NT—neural tube, Som—somites. (**E**) Higher magnification of box denoted in C, arrows indicate somites, fl—forelimb. (**F**) Magnification of box shown in C. Dotted line demarcates the apical ectodermal ridge (AER), fl—forelimb.

Very little is known about the wider developmental role of Stat3 in mammals following gastrulation as *Stat3* null animals fail to progress beyond this critical stage [26]. To establish which tissues potentially require Stat3 functionality post-gastrulation, and more specifically if Stat3 is relevant to early bone development, we performed *in situ* hybridization analysis of *Stat3* mRNA localization at various early stages following germ layer specification. Similar to a published report in zebrafish [28], at E8.5, *Stat3* mRNA is manifest within the anterior mesenchyme as well as within the somites and posterior presomitic mesoderm (Fig 1A and 1B and S1A and S1B Fig). At the more developed stage of E10.5, *Stat3* expression is observed clearly in all somites (Fig 1C and 1E and S1C and S1E Fig). Further, *Stat3* expression is especially prominent within the limb bud mesoderm but is absent or greatly diminished within the apical ectodermal ridge (AER) adjacent to this zone of expression (Fig 1C and 1F and S1C and S1F Fig). Upon transverse sectioning at the level of the hindlimb, *Stat3* expression at E10.5 is generally weak, though specific compared to control, throughout the limb bud, sclerotome and especially myotome (Fig 1D and S1D Fig). Interestingly, a modestly heightened level of *Stat3* expression is evident within the ventral-lateral neural tube, suggesting this to be the area of dominant antero-posterior staining within the whole mount analysis (Fig 1C–1E and S1C–S1E Fig). Overall, these findings suggest a potential post-gastrulation role for Stat3, specifically in the development of mesoderm-derived tissues.

### Mesodermal *Stat3* facilitates post-natal skeletogenesis

**Fig 2:**
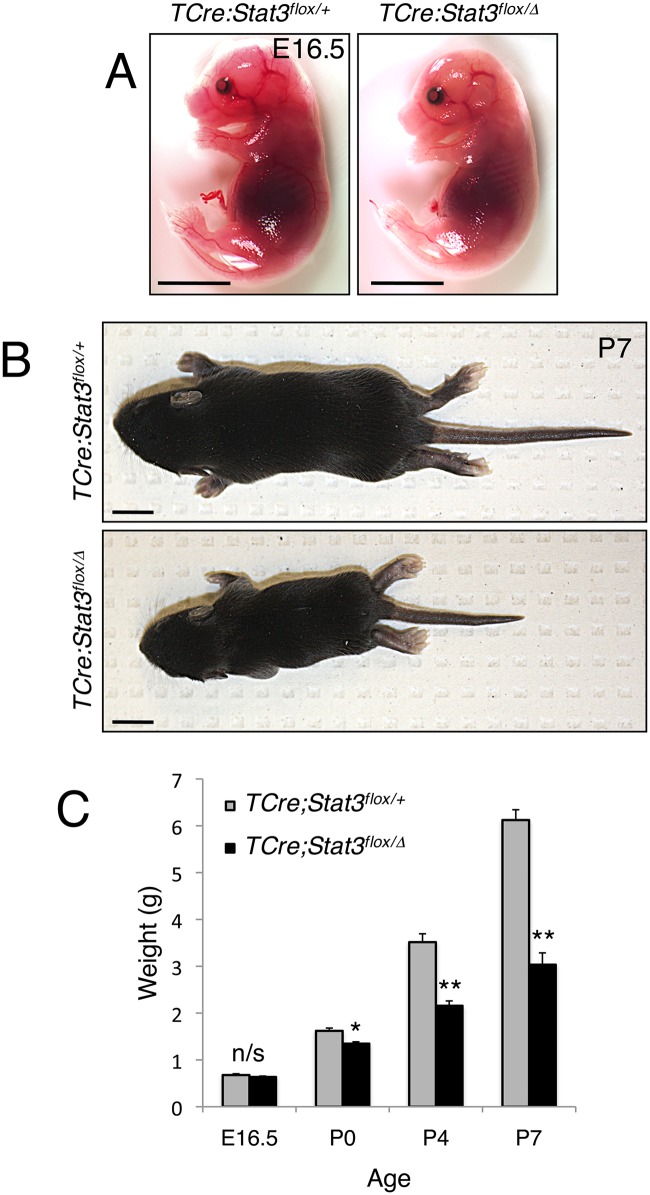
Abnormal postnatal growth of *Stat3*-deficient mice. (**A** and **B**) Representative images of littermate matched control and *TCre;Stat3*^*flox/Δ*^ mutant mice at E16.5 and P7, respectively. Bar = 1cm. (**C**) Chart depicting average weights of indicated genotypes in aging mice. Error bars denote the standard error of the mean (SEM), n/s—not significant, *p<0.05, **p<0.01.

Since germline deletion of *Stat3* results in early embryonic lethality [26], we generated mice conditionally lacking Stat3 expression in essentially all mesodermal tissues by crossing mice carrying the *Stat3* floxed allele (*Stat3*^*flox/flox*^) with a homozygous T-Cre driver strain, heterozygous for *Stat3* (*TCre;Stat3*^*Δ/+*^) [29, 30]. *TCre;Stat3*^*flox/Δ*^ (mutant) animals were generated at the expected Mendelian ratio of 50% and were compared to *TCre;Stat3*^*flox/+*^ (control) littermates in all subsequent analyses (S1 Table). Importantly, Stat3 protein levels are ablated in all mutant mesoderm-derived neonatal tissues (protein extract from P0 humeri shown), confirming effective deletion of the functional gene product (S2A Fig). *TCre;Stat3*^*flox/Δ*^ mutants were virtually indistinguishable from controls late in gestation (E16.5) and, at birth (P0) displayed only a modest but significant reduction in bodyweight (Fig 2A and 2C). At P4, *TCre;Stat3*^*flox/Δ*^ mutants became easily identifiable, exhibiting antero-posterior shortening of the body axis and reduction in body weight (Fig 2C). By P7, both the body axis defect and body weight disparity of the *TCre;Stat3*^*flox/Δ*^ animals was more pronounced (Fig 2B and 2C). Further, between P4 and P7, *TCre;Stat3*^*flox/Δ*^ animals developed progressive respiratory distress and likely succumbed to a multiplicity of defects including a lack of mobility, labored breathing and failure to thrive during this period, though the specific cause of death was not investigated. Interestingly, at P4 and P7, *TCre;Stat3*^*flox/Δ*^ mutants had difficulty ambulating and displayed an obvious abnormal curvature of both the forelimbs and hindlimbs, a defect which was exacerbated in the few animals surviving past P7 (S2B Fig).

To address the most obvious gross defect of the *TCre;Stat3*^*flox/Δ*^ animals, i.e., the dramatic antero-posterior body axis reduction (Fig 2B), we performed whole mount *in situ* mRNA hybridizations in *TCre;Stat3*^*flox/Δ*^ and littermate control embryos to determine whether somitogenesis and/or segmentation are directly affected by *Stat3* deletion. *TCre;Stat3*^*flox/Δ*^ mutant embryos formed a full complement of normal, polarized somites based on the expression pattern at E12.5 for myogenin (*MyoG*), a marker of skeletal muscle differentiation, and scleraxis (*Scx*), which labels the emergent tendon precursors, being no different in *TCre;Stat3*^*flox/Δ*^ mutants relative to control embryos (S3A and S3B Fig). Further, analysis of multiple aged *TCre;Stat3*^*flox/Δ*^ mutant animals (n>24) demonstrated a complete set of vertebrae including the typical 7 cervical, 13 thoracic, 6 lumbar, 4 sacral and a variable but normal range of caudal vertebrae. Together, these data suggest that the antero-posterior axis defect is not a result of disrupted axis extension or specification.

**Fig 3:**
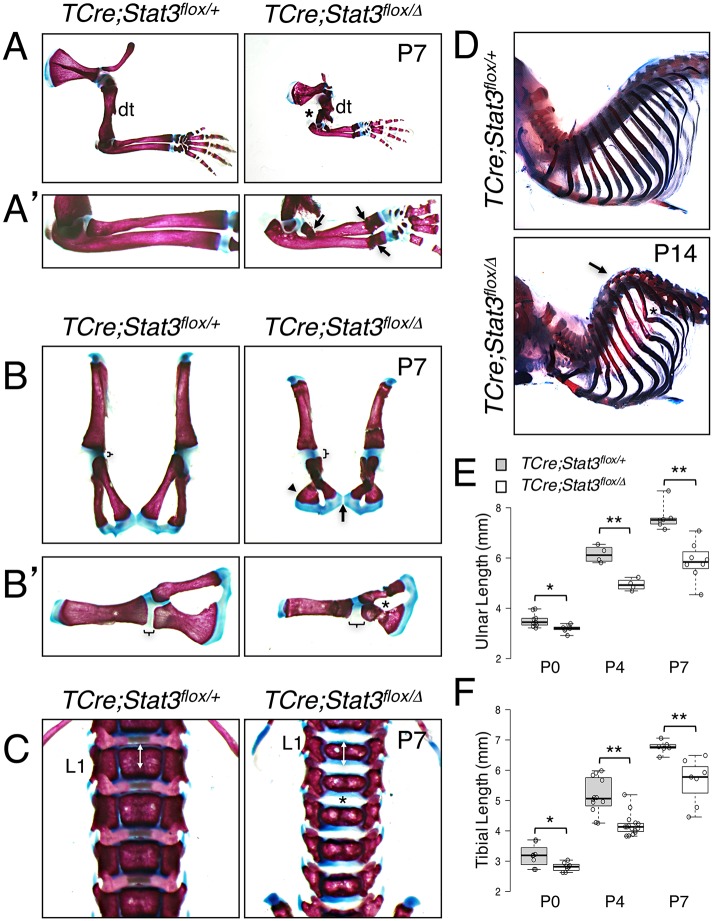
Abnormal skeletogenesis in *TCre;Stat3*^*flox/Δ*^ mice. (**A** and **A’**) Alizarin Red/Alcian Blue-stained skeletal preparations demonstrating bending and spontaneous fracture of forelimbs in *TCre;Stat3*^*flox/Δ*^ mice. Asterisk indicates humerus, arrows indicate fractures of radius/ulna, dt—deltoid tuberosity. (**B** and **B’**) Alizarin Red/Alcian Blue-stained skeletal preparations depicting dysplastic hip girdles in *TCre;Stat3*^*flox/Δ*^ mice. Brackets denote acetabular cartilage; arrow indicates pubic symphysis; arrowhead indicates ischium; asterisk denotes spontaneous fracture of pubis bone. (**C**) Alizarin Red/Alcian Blue-stained skeletal preparations demonstrating antero-posterior compression of vertebral elements in *TCre;Stat3*^*flox/Δ*^ mice. Double arrows indicate length of mutant vertebral body, asterisk indicates intervertebral space, L1—1^st^ lumbar vertebrae. (**D**) Alizarin Red/Alcian Blue-stained skeletal preparations demonstrating spinal kyphosis in *TCre;Stat3*^*flox/Δ*^ mice. Arrow indicates abnormally curved spine, asterisk indicates caudal rib element fracture. (**E** and **F**) Box-and-whisker plots for ulnar (**E**) and tibial (**F**) lengths (mm). Error bars represent SEM, *p<0.05, **p<0.01.

We then analyzed whole skeletal preparations to determine if the phenotype is a result of a more generalized bone defect. We observed a range of skeletal flaws, which in some cases were evident as early as P0 and became progressively more pronounced over time. Appendicular defects contributing to the limb phenotype included bent elements of the limbs, most notably in the radii, and spontaneous fractures (Fig 3A and 3A’ and S3A and S3B Fig).

*TCre;Stat3*^*flox/Δ*^ mutant scapulae became misshapen with advancing postnatal age, and humeri were often observed with mild to severe fractures (Fig 3A and S3A and S3B Fig). Further, *TCre;Stat3*^*flox/Δ*^ mutant hip girdles became progressively dysplastic, having laterally flared ilia and ischia, abnormal ventral curvatures of the pubis region, and frequent spontaneous fractures (Fig 3B and S3C and S3D Fig). We also observed apparent changes in the cartilaginous regions of the hip girdle, most pronounced at P7, where mutant animals exhibited an antero-posterior extension of cartilage around the acetabulum and a poorly fused pubic symphysis (Fig 3B, arrow and Fig 3B’). An overall consistent and significant shortening of mutant long bones from both the forelimb and hindlimb suggests a post-natal defect in cartilage template development during element maturation (Fig 3E and 3F and S4G Fig). To examine this further, we looked at the elements of the axial skeleton, which are also formed via a cartilage template-driven process. From P0 through P7, the individual vertebral elements of *TCre;Stat3*^*flox/Δ*^ mutant mice maintain a fairly normal lateral dimension but fail to elongate along the antero-posterior growth axis (Fig 3C and S3E and S3F Fig). This growth defect is accompanied by an expansion of the intervertebral space and reduced or absent intervertebral discs (Fig 3C and S3E and S3F Fig). Finally, we observed a progressive kyphosis of the mid-thoracic spine, which is evident as early as P4 and striking in P14 animals (Fig 3D). This curvature resulted in an abnormal posterior rib arrangement and likely led to the observed fractures of multiple caudal rib elements (Fig 3D). In total, *TCre;Stat3*^*flox/Δ*^ mutant animals suffer from a failure to properly form endochondral bones, leading to a premature cessation of elongation in both the appendicular and axial skeleton and subsequent dwarfism and skeletal distortion.

### Stat3 regulates chondrocyte hypertrophy in the growth plate

**Fig 4:**
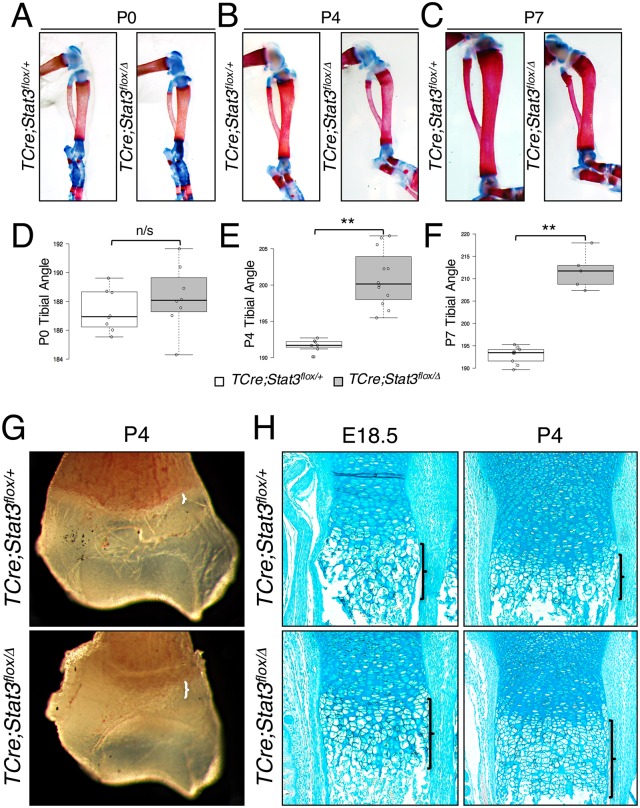
Tibial bowing and disruption of endochondral ossification in *TCre;Stat3*^*flox/Δ*^ mice. (**A**-**C**) Alizarin Red/Alcian Blue-stained skeletal preparations demonstrating progressive angulation of tibiae in*TCre;Stat3*^*flox/Δ*^ mice. (**D**-**F**) Box-and-whisker plots quantifying angle of tibiae for control and mutant animals in A-C. Error bars represent SEM, n/s—not significant, **p<0.01. (**G**) Whole mount brightfield image of distal humeri. Brackets indicate width of hypertrophic chondrocyte zone. (**H**) Alcian Blue-stained longitudinal sections of the distal ulnae of control and mutant *TCre;Stat3*^*flox/Δ*^ mice at indicated ages. Brackets denote the length of the hypertrophic chondrocyte region.

As many of the *Stat3* mutant long bones appear misshapen but seem to fracture early, we more specifically addressed frank bowing of the long bones in control and mutant tibial elements. These bones withstood the incidence of fracture for a longer period postnatally in our mutant animals. Measurements were made as shown in S5 Fig. At birth, we observed a slight increase in angulation of *TCre;Stat3*^*flox/Δ*^ mutants at the tibial crest (Fig 4A and 4D) though this was not significant. However, at P4 (Fig 4B and 4E) and dramatically at P7 (Fig 4C and 4F and S5A and S5B Fig), there was a distinct and highly significant bowing of *TCre;Stat3*^*flox/Δ*^ mutant tibiae prior to the onset of incident fractures that were more prevalent by P7.

It has been reported that Stat3 functions in the regulation of both osteoclast and osteoblast activities to modulate bone resorption and mineralization respectively [31–33]; however, our *TCre;Stat3*^*flox/Δ*^ mutant mice exhibit an earlier and more severe bone phenotype than previously described, suggesting a possible role for Stat3 upstream of osteoblast lineage commitment. It has further been established that Stat3 is present in growth plate chondrocytes and plays a pro-proliferative role [34]. We here confirmed the presence of Stat3 in order to make potential inferences about its loss in our mutant mouse model. In normal E14.5 humeri, we observe Stat3 expression in the preponderance of chondrocytes within the epiphysis (S6A Fig). This observation is supported by immunoblot analysis of lysates created from dissected epiphyseal regions of control P0 and P4 humeri, and fortified by the absence of Stat3 in *TCre;Stat3*^*flox/Δ*^ mutant littermate epiphyseal lysates (S6B, S6C and S6D Fig). These data support established literature and suggest that a disruption of Stat3 within the earliest bone-forming cells could reasonably have a biological impact.

To determine if the observed skeletal phenotype could be attributed to a combinatorial effect of targeting both the osteochondro progenitor pool and monocyte/osteoclast lineage, we created conditional *Stat3* loss-of-function animals by crossing mice carrying the *Stat3* floxed allele with the *Prx1Cre* driver mouse strain [35], which would only target the osteochondro progenitor lineage, and most completely in the forelimb. *Prx1Cre;Stat3*^*flox/Δ*^ mutant animals were born at the expected Mendelian ratio and were phenotypically indistinguishable from littermate controls at birth (S1 Table and S7A and S7C Fig). At P4, however, *Prx1Cre;Stat3*^*flox/Δ*^ animals exhibited a modest reduction in body weight relative to controls and appeared to phenocopy the curvature of forelimbs observed in the *TCre;Stat3*^*flox/Δ*^ mutants (S7B and S7C Fig). Additionally, *Prx1Cre;Stat3*^*flox/Δ*^ mutant skeletal preparations revealed a similar bent morphology of their radii and ulnae likely the result of co-incident fractures, and more strikingly, a fully penetrant incidence of bi-lateral mid-shaft fractures of the humeri (S7D and S7E Fig). These findings suggest that the limb defects in *Stat3* loss-of-function mutants are independent of direct effects within the osteoclast pool, as recombination mediated by *Prx1Cre* should not affect the monocyte lineage, the source of osteoclast precursors [36].

We next evaluated the histological features of the forelimb long bones in our two *Stat3* loss-of-function mutant lines. Initially, whole mount analysis of fixed humeri from *TCre;Stat3*^*flox/Δ*^ animals at P4 revealed a region of opacity at the edge of the presumptive growth plate that appeared markedly wider in the long bones of the *TCre;Stat3*^*flox/Δ*^ mutant animals (Fig 4G). We analyzed this region in ulnae by alcian blue (aggrecan) staining at various ages and found that the zone of chondrocyte hypertrophy was extended in the *TCre;Stat3*^*flox/Δ*^ mutants as early as E18.5 and that this imbalance was even more dramatic at P4 (Fig 4H). Strikingly, though unsurprisingly as Stat3 is known to regulate osteoblast maturation and function, *TCre;Stat3*^*flox/Δ*^mutant long bones also demonstrated a reduction in trabecular bone (Fig 4H)[31, 33]. As confirmation of these phenotypes, we saw a similar extension of the hypertrophic zone in *Prx1Cre;Stat3*^*flox/Δ*^ mutant long bones (humeri) of the forelimb (S7F and S7G Fig), again indicating the phenotype is specific and independent of Stat3’s function within the osteoclast population. Further, *Prx1Cre;Stat3*^*flox/Δ*^ mutants also exhibit a similar decrease in trabecular bone associated with the loss of Stat3 (S7F and S7G Fig).

### *Stat3* loss-of-function mimics features of *Sox9* haploinsufficiency

**Fig 5:**
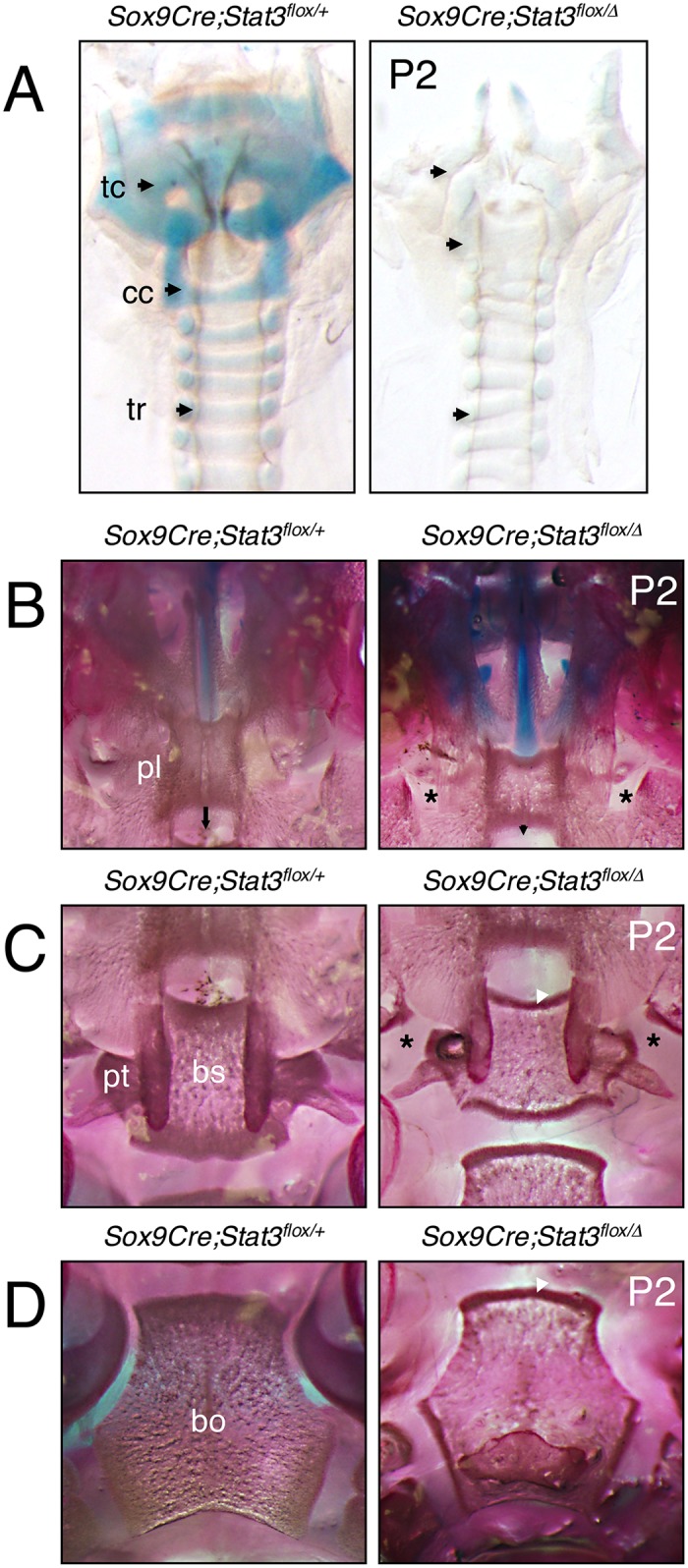
Tracheal and palatal malformations in *Sox9Cre;Stat3*^*flox/Δ*^ mice. (**A**) Whole mount Alcian Blue-stained larynx and trachea from P2 control and *Sox9Cre;Stat3*^*flox/Δ*^ mice. Arrows correspond to indicated cartilage regions, tc—thyroid cartilage, cc—cricoid cartilage, tr—tracheal ring cartilage. (**B**—**D**) Ventral aspect of Alizarin Red/Alcian Blue stained palates in control and *Sox9Cre;Stat3*^*flox/Δ*^ mice. Arrows indicate caudal extension of secondary palate, asterisks indicate absence of palatal bone fusions, white arrowheads indicate osteosclerotic regions. pl—secondary palate, pt—pterygoid bone, bs—basisphenoid bone, bo—basioccipital bone.

Our observations to this point are strongly reminiscent of many elements of the phenotype reported for the *Sox9*^*+/-*^ haploinsufficient mouse model, including bent limbs, dwarfism, hip dysplasia, spinal curvature, extension of the hypertrophic chondrocyte zone and perinatal lethality [7]. To investigate additional features i*n vivo* associated with *Sox9* haploinsufficiency in tissues where *TCre* is not known to be active, we employed a *Sox9Cre* driver strain strategy [37]. *Sox9Cre;Stat3*^*flox/Δ*^ mutants suffer from a heightened incidence of perinatal lethality, as neonates were produced at an observed rate of 8.3% (n = 4/48) versus an expected rate of 25% (n = 12/48) based on the genetic cross used (S1 Table). Furthermore, pups surviving the gestational period had severely labored breathing and died within 48 hours of birth. We analyzed specific regions of the *Sox9Cre;Stat3*^*flox/Δ*^ animals for further evidence of phenotype reminiscent of *Sox9* hapoinsufficiency and found that the thyroid, cricoid and tracheal ring cartilages associated with the developing larynx and trachea were markedly reduced compared to littermate controls (Fig 5A). Within the developing palates of the *Sox9Cre;Stat3*^*flox/Δ*^ mutants at P2, we observed a failure of the secondary palate to extend caudally and laterally, resulting in a lack of proper fusion (Fig 5B). This open palate persists caudally around the basisphenoid bone, pterygoid processes and the basioccipital bone, coupled with obvious osteosclerosis (arrowheads) adjacent to the presumptive growth plates of these developing bones (Fig 5C and 5D). The composite phenotypes observed across our series of mutant animals offer corroborating evidence in support of a role for Stat3 in the pathology of *Sox9* haploinsufficiency-like defects, including palate closure defects and laryngotracheomalacia. A comparison of the similar, and distinct, features of our series of loss-of-function mutant mice with the *Sox9*^*+/-*^ model and human CD is summarized in Table 1.

**Table 1:** Comparison of phenotypic abnormalities in Stat3 loss-of-function mice relative to Sox9 haploinsufficient mice and patients with campomelic dysplasia. Presence or absence of phenoypte denoted as + or -, respectively. Phenotype not investigated denoted as “N.I.”. Phenotypic information not available denoted as “unreported”.

Feature	Patients with CD	*Sox9+/-* Mouse	*TCre* Mutant	*Prx1Cre* Mutant	*Sox9Cre* Mutant
Early Death	+	+	+	N.I.	+
Dwarfism	+	Unreported	+	+	N.I.
Respiratory distress	+	+	+	-	+
Cleft Palate	+	+	+	N.I.	+
Laryngotracheomalacia	+	+	+	N.I.	+
Small thoracic cage	+	+	N.I.	N.I.	N.I.
Micrognathia	+	+	N.I.	N.I.	N.I.
Bowing of long bones	+	+	+	+	N.I.
Bowed tibiae	+	+	+	N.I.	N.I.
Bowed femora	+	-	-	N.I.	N.I.
Bowed ulnae or radii	+	+	*+	*+	N.I.
Fracture of long bones	-	-	+	+	N.I.
Trabecular reduction	-	-	+	+	N.I.
Pelvic deformity	+	+	+	N.I.	N.I.
Spinal deformity	+	+	+	N.I.	N.I.
Missing pair of ribs	+	-	-	-	-
XY sex reversal	+	-	**	**	N.I.
Hypertrophic zone elongation	Unreported	+	+	+	N.I.

*Analysis obscured by co-incident fracture.

**Indicated Cre inactive in tissue of origin.

### Loss of *Stat3* deregulates *Sox9 in vivo*

**Fig 6:**
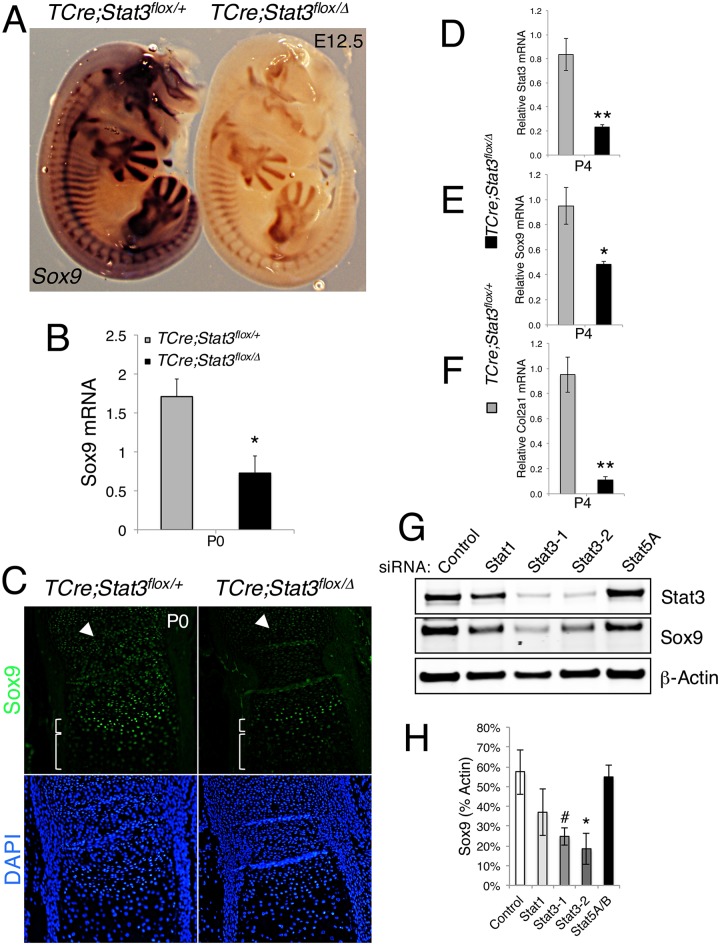
Sox9 is deregulated *in vivo* in *TCre;Stat3*^*flox/Δ*^ mice. (**A**) WISH for *Sox9* expression in E12.5 embryos of indicated genotype. (**B**) Total RNA isolated from humeri of control and mutant *TCre;Stat3*^*flox/Δ*^ mice at birth analyzed for *Sox9* expression by quantitative RT-PCR. Error bars represent SEM, *p<0.05. (**C**) Sox9 protein levels analyzed by immunofluorescence in longitudinal sections of the distal ulnae of control and mutant *TCre;Stat3*^*flox/Δ*^ mice at birth. Arrowhead marks resting chondrocytes, small bracket highlights prehypertrophic chondrocytes, large bracket denotes hypertrophic zone. (**D**-**F**) Total RNA isolated from humeri of control and mutant *TCre;Stat3*^*flox/Δ*^ mice at birth analyzed for (**D**) *Stat3*, (**E**) *Sox9 and* (**F**) *Col2a1* expression by quantitative RT-PCR. Error bars represent SEM, *p<0.05, **p<0.01. (**G**) Representative immunoblot analysis of indicated proteins in response to Stat family member siRNA constructs after 72h. (**H**) Quantitative analysis of Sox9 levels from immunoblot in G, error bars are SEM, #p = 0.054, *p<0.05.

To date, mutations or deletions of the *SOX9* locus are the only known genetic lesions responsible for development of CD in humans. As previously noted, the composite phenotypes of our *Stat3* loss-of-function models mimic the defects reported in the *Sox9*^*+/-*^ mouse model [7]. In order to determine whether Stat3 could potentially act through the regulation of *Sox9*, we investigated *Sox9* expression and function in our *TCre;Stat3*^*flox/Δ*^ model. At mid-gestation, *TCre;Stat3*^*flox/Δ*^ mutant embryos display a spatially normal, but global reduction in *Sox9* expression compared to littermate controls as determined by *in situ* hybridization (Fig 6A). Importantly, as the characterization of our *Cre* driver had not been evaluated as late as embryonic day 12.5, we undertook a *TCre* lineage trace by crossing male drivers to female *Rosa*^*LacZ/+*^ animals and evaluated β-galactosidase activity on similarly staged embryos [38]. Brief exposure to substrate indicated anterior areas where *TCre* demonstrates weak recombination activity (S8A Fig). However, longer exposure to substrate revealed extensive and specific patterns of recombination in areas consistent with cranial neural crest migratory routes when compared to controls (S8B–S8E Fig). We observed on a macroscopic level, recombination in the dorsal anterior neural tube, the source for cranial neural crest cells, indicating a high likelihood that anterior areas of *Sox9* reduction could indeed be a cell autonomous response to the loss of *Stat3* (S8D Fig). We cannot, however, completely rule out a non-cell autonomous phenomenon in the anterior portions of our *TCre* mutants, leaving open the possibility that some effects are indirectly linked to the loss of *Stat3*.

To quantitatively measure the observed change in *Sox9* mRNA, we performed quantitative RT-PCR (RT-qPCR) for *Sox9* from total RNA isolated from the humeri of individual P0 *TCre;Stat3*^*flox/Δ*^ mutant and littermate controls and found a significant reduction in *Sox9* expression, confirming our *in situ* results (Fig 6B). We next examined if this reduction in mRNA translates to a decrease in Sox9 protein, using immunofluorescent analysis for Sox9 on neonatal sections of radii, and found that in *TCre;Stat3*^*flox/Δ*^ mutants, there is an overall decrease in the amount of Sox9 staining throughout the epiphysis and growth plate chondrocytes (Fig 6C). Finally, we assessed the physiological relevance of the observed reduction in Sox9 by evaluating the expression of an early Sox9 direct target, *type II collagen* (*Col2α1*), by RT-qPCR. As expected, mutant mice exhibited a predictable decrease in *Stat3* as well as *Sox9* mRNA at P4, when the observed phenotype becomes quite evident (Fig 6D and 6E). Importantly, a concomitant reduction in the expression of *Col2α1* in our *TCre;Stat3*^*flox/Δ*^ mutants was also observed, suggesting that Sox9 levels had fallen beneath a threshold required for adequate chondrocyte function (Fig 6F). These findings indicate that the loss of Stat3 in osteochondro progenitors deregulates the functional complement of Sox9, implying a potential mechanism whereby our observed skeletal defects may arise.

In an effort to validate our *in vivo* observations in an *ex vivo* setting, we used a primary limb bud cell culture model derived from E13.5 F344 rat embryos, a stage analogous to E11.5 in the mouse (S9A and S9B Fig). Here, we tested transient (72h) siRNA-induced knockdown of individual *Stat* family members in these cells to address the early effect of *Stat* loss-of-function on Sox9 levels. We found that knockdown of *Stat3*, by two independent siRNA constructs, significantly reduces Sox9 protein levels compared to control siRNA, whereas this effect is not observed when either *Stat1* or *Stat5A* are knocked down, suggesting a Stat3-specific phenomenon (Fig 6G and 6H). Evidence using cell culture models suggests that fibroblast growth factor (Fgf) signaling through extracellular regulated kinase 1/2 (ERK1/2) may regulate the expression of Sox9 [39]. Importantly, knockdown of *Stat3* does not negatively affect MAPK signaling as measured by pERK1/2 levels, suggesting that endogenous levels of Stat3, specifically, are required to maintain normal Sox9 levels (Fig 6G and 6H and S9D and S9E Fig). Interestingly, knockdown of *Stat1*, but not *Stat5*, negatively affects activated MAPK levels; however, in either case there is no significant change in Sox9, further supporting a regulatory phenomenon likely specific to Stat3 (Fig 6G and 6H and S9D and S9E Fig).

### Stat3 likely regulates *Sox9* expression directly *ex vivo*

**Fig 7:**
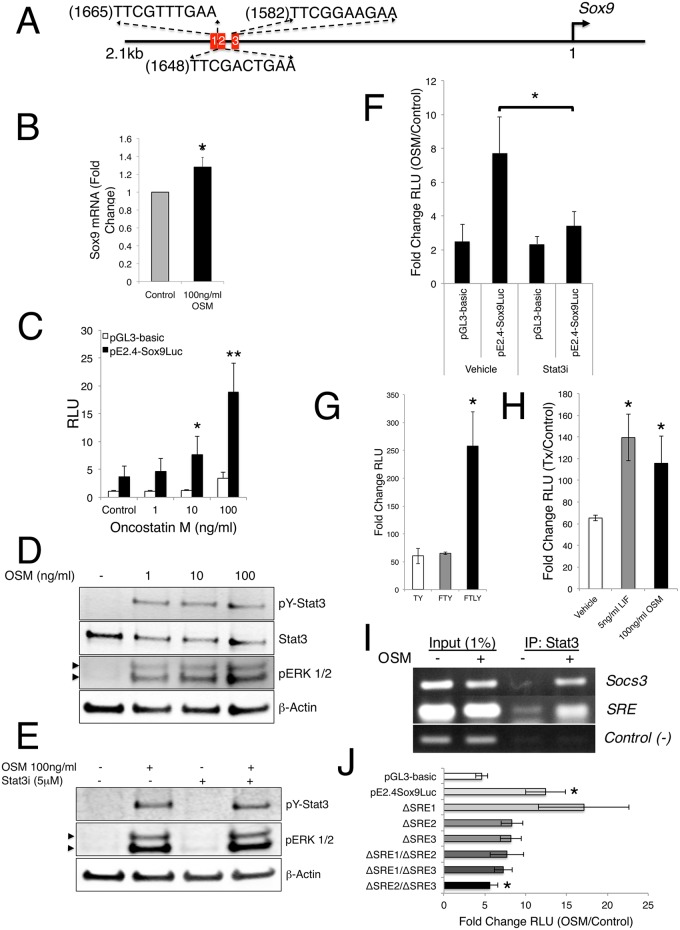
Sox9 expression is regulated by Stat3. (**A**) Schematic of immediate upstream mouse *Sox9* locus. Red blocks indicate putative Stat DNA binding elements. (**B**) Total RNA isolated from control or OSM-treated 3T3 cells analyzed for *Sox9* expression by quantitative RT-PCR after 30 minutes. Error bars represent SEM, *p<0.05. (**C**) Analysis in 3T3 cells of a Sox9 promoter-driven luciferase construct in response to control or OSM treatment for 24h at specified doses. Error bars are SEM, *p<0.05, **p<0.01. (**D**) Immunoblot analysis depicting activation of indicated proteins in response to increasing OSM levels in 3T3 cells. Arrowheads indicate doublet isoforms of ERK. (**E**) Immunoblot analysis depicting activation of indicated proteins in response to OSM treatment in the presence of a Stat3 inhibitor (Stat3i) in 3T3 cells. Arrowheads indicate doublet isoforms of ERK. (**F**) Fold change analysis of a *Sox9* promoter-driven luciferase construct in response to OSM treatment in the presence of a Stat3i for 24h in 3T3 cells. Error bars are SEM, *p<0.05. (**G**) Analysis of a *Sox9* promoter-driven luciferase construct in response to indicated treatment for 48h in rat limb bud cells. Error bars are SEM, *p<0.05. (**H**) Analysis of a *Sox9* promoter-driven luciferase construct in response to indicated treatment for 24h in growth conditionsFgf2 (50ng/ml)/TGFα (10ng/ml)/Y27632 (10μM) (FTY) media. Error bars are SEM, *p<0.05. (**I**) Chromatin immunoprecipitation analysis of DNA associated with Stat3 protein in response to OSM treatment in 3T3 cells by PCR for specified regions. (**J**) Fold change analysis of indicated Sox9 promoter-driven luciferase constructs mutated in the Stat binding regions in response to OSM treatment in 3T3 cells for 24h. Error bars are SEM, *p<0.05.

Very little is known about signals that potentiate the expression of Sox9 *in vivo* leading us to inquire about the mechanism by which loss of *Stat3* achieves the observed changes in *Sox9*. We addressed this question by first examining the 2.1 Kb immediate upstream promoter region of *Sox9 in silico* to identify potential Stat-specific DNA response elements (SRE) corresponding to the consensus sequence, 5’-TTC(N_3-4_)GAA-3’, and found three clustered SREs at 1665 (5’-TTCGTTTGAA), 1648 (5’-TTCGACTGAA-3’) bases and 1582 (5’-TTCGGAAGAA-3’) bases upstream of the transcriptional start site, the first of which was of lower confidence than the other two (Fig 7A). Additionally, we observed a single, low-confidence SRE at 674 (5’-TTCGTTGAA-3’) bases upstream of the start site (not depicted). It is reasonably well established that the promoter region of *Sox9* is only modestly conserved. To determine if our observation was limited to the mouse, we extended our promoter evaluation into *Drosophila*, rat, and human. We found that, while the specific sequence and precise position(s) of identified Stat consensus sites were variable, all species evaluated contained at least one putative Stat-binding site at approximately 1.6Kb upstream of the *Sox9* ortholog transcriptional start site (S10A Fig). Taking a conservative view of the probability of any 10-mer DNA sequence appearing at random over a region of 2.1Kb, the appearance of these Stat-binding sites anywhere in the upstream promoter is highly improbable (0.04%), suggesting their specific upstream location is unlikely to be a random event and has potential biological significance.

Since *in vivo* deletion of either *LIF* or *LIFR* does not result in alteration of the hypertrophic chondrocyte zone [40, 41], we next asked if other potentially relevant initiators of Stat3 activation in the IL-6 family of cytokines to which LIF belongs, e.g., IL-6 or Oncostatin-M (OSM), could regulate *Sox9* expression in cell culture. We first addressed this question using a NIH3T3 mouse fibroblast culture model. These cells mimic many features of early mesenchymal cells and have been shown to undergo BMP4-induced chondrogenic lineage commitment in culture and contribute to robust collagen formation *in vivo* [42]. These cells express nuclear Sox9 and rapidly facilitate translocation of Stat3 to the nucleus in response to ligand stimulation (S11A Fig). By RT-qPCR analysis, we determined that both OSM and IL-6 rapidly (30 minutes) induce a modest but significant upregulation of *Sox9* mRNA in these cells (Fig 7B and S11B Fig). Additionally, we demonstrated that a previously described mouse 2.4kb *Sox9* promoter-driven luciferase reporter construct [43], which contains the putative SRE sites, is induced in a dose-dependent fashion by OSM in culture (Fig 7C).

In addition to activating Stat3 and other Stat family members, IL-6 and OSM can also induce phosphorylation of ERK1/2 to regulate downstream transcriptional targets. As previously mentioned, ERK1/2 is a suspected modulator of Sox9 in cell culture. To assess the ability of these ligands to initiate both signaling pathways, we analyzed 3T3 cells challenged for 20 minutes with increasing doses of either IL-6 or OSM by immunoblotting. IL-6 induced phosphorylation and activation of Stat3 in a dose-dependent manner, but did not activate ERK1/2, Stat1, or Stat5, suggesting the modest increases in *Sox9* expression and promoter activity are mediated primarily by Stat3 (S11C and S11D Fig). However, OSM robustly activated Stat3 as well as Stat1, Stat5, and ERK1/2, suggesting that OSM-induced *Sox9* promoter activity may not be solely attributable to Stat3 signaling (Fig 7D and S11D Fig). To test this directly, we analyzed 3T3 cells transfected with the *Sox9* luciferase reporter and co-treated with a specific peptide-inhibitor for Stat3 (Stat3i), which targets transcriptional activation [44]. As expected Stat3 was still phosphorylated in response to OSM (Fig 7E); however, we found that the specific inhibition by Stat3i dramatically abrogated the ability of OSM to induce *Sox9* promoter-driven luciferase activity above baseline levels (Fig 7F). Importantly, the addition of Stat3i to our culture system did not affect the phosphorylation of ERK1/2 in response to OSM stimulation (Fig 7E), suggesting that the inhibition of Stat3 was both specific and responsible for the decrease in OSM-induced *Sox9* promoter-driven luciferase activity.

To confirm that our observations were not due to a cell-specific phenomenon, we again utilized our rat primary limb bud cell culture *ex vivo* model. Importantly, these cells respond similarly to either OSM or LIF as measured by the rapid (20 minutes) phosphorylation of Stat3 (S9C Fig), suggesting the availability of multiple receptors capable of transducing the JAK/Stat signal. We investigated how different growth conditions, including Tgf-α and ROCK inhibitor, Y27632 (TY), with the addition of Fgf-2 (FTY) or Fgf-2 and LIF (FTLY), affect the basal activity of our pE2.4Sox9-luciferase reporter and found that the addition of LIF facilitates a significant increase in luciferase activity (Fig 7G). Further, we found that cells cultured in FTY conditions, but not in TY or FTLY conditions, are amenable to stimulation by either OSM or LIF, in turn significantly upregulating the activity of the *Sox9* driven luciferase reporter (Fig 7H and S11E and S11F Fig) and confirming the requirement for Fgf signaling to maintain *Sox9* inducibility as previously reported [45]. In total, these data validate our findings in NIH3T3 cells, provide additional evidence in a second species of the requirement for Stat3 in maintaining normal levels of *Sox9*, and demonstrate that minimally three upstream ligands (IL-6, OSM and LIF) can mediate these effects in cell culture and are potentially relevant *in vivo*.

Our findings to this point strongly suggested a *bona fide* function for Stat3 in modulating the expression of *Sox9* in a cell culture setting, though the question of whether Stat3 achieves this directly remained open. To address this question, we first created a *Sox9* promoter-driven luciferase construct wherein the upstream 1630 base pairs were deleted, removing all four of the identified SREs (pE770Sox9-Luc) and tested it in 3T3 cells. Kanai et al., previously demonstrated that deletion of the longer-range elements more than 200bp upstream of the Sox9-luciferase reporter does not affect reporter activity after growth in serum in multiple cell culture models [43]. We found that the activity of this deletion construct was not significantly enhanced by stimulation with OSM following serum starvation and that the modest increase in activity was not significantly altered by the addition of the Stat3i peptide (S11G Fig). This finding suggests that the removal of the SREs abrogates the ability of OSM to induce *Sox9* promoter activity; however, it does not preclude the notion that other elements within the large deleted region may be responsive to factors other than Stat proteins.

To address the possibility that Stat3 is directly responsible for driving *Sox9* expression in cell culture, we performed chromatin immunoprecipitation (ChIP) analysis on the putative regions of *Sox9* promoter DNA specifically associated with Stat3 in OSM-treated NIH3T3 cells. As expected, OSM treatment induced recruitment of Stat3 to the *Socs3* gene when compared to untreated cells [46]. This served as a positive control for our analyses (Fig 7I). We also detected a robust recruitment of Stat3 specifically to the region around the upstream cluster of SREs within the *Sox9* promoter following OSM treatment, though assessment of Stat3 occupancy of specific individual SRE sites was precluded due to their close proximity to one another and the average DNA fragmentation size (~500bp) used in our experimental conditions (Fig 7I). Importantly, analysis of the fourth SRE site, roughly 950bp downstream of the SRE cluster, demonstrated that Stat3 was not recruited to a quite proximal area under the same conditions and therefore served as a negative control in establishing the specificity of the upstream Stat3/DNA association (Fig 7I). These results indicate a likely physical interaction between Stat3 and the *Sox9* promoter in response to Stat3 activation, at least in cell culture. Further, ChIP analyses would suggest that Stat3 is directly activating the expression of *Sox9*, but do not precisely define the binding site(s) within the promoter.

To investigate which of the upstream SRE elements Stat3 binds to, we generated a series of mutant pE2.4Sox9-luciferase constructs where the 5’ palindromic sequence of each individual SRE was altered from “TTC” to “CCG” to abrogate the ability of Stat3 to effectively bind the DNA (pΔSRE1Sox9-Luc, pΔSRE2Sox9-Luc and pΔSRE3Sox9-Luc). When tested in our 3T3 cell culture model, the individual mutants do not significantly alter reporter activity (Fig 7J). As oligomerization of Stats often enhances their transcriptional activity, we also made and investigated tandem SRE site pE2.4Sox9-luciferase mutants in 3T3 cells (pΔSRE1/ΔSRE2Sox9-Luc, pΔSRE1/ΔSRE3Sox9-Luc and pΔ2SRE/ΔSRE3Sox9-Luc). When SRE2 or SRE3 were mutated in tandem with SRE1, we again observed no significant alteration of luciferase activity in response to stimulation by OSM (Fig 7J). However, when SRE2 and SRE3 were mutated in tandem we observed a significant decrease in the amount of reporter activity as compared to the wild-type pE2.4Sox9-luciferase in response to OSM stimulation (Fig 7J). These findings validate our ChIP analysis, effectively placing Stat3 directly on SRE2 and/or SRE3, and suggest these sites are required for the regulation of *Sox9* by JAK/Stat signaling through Stat3 in cell culture, a mechanism we suggest is likely operant for proper skeletogenesis *in vivo*, though this remains an open question.

## Discussion

The current study defines an as yet unappreciated function for Stat3 in mammalian embryonic development following germ-layer formation at gastrulation. Specifically, mesodermal loss of *Stat3* causes early neonatal lethality and a dramatic skeletal dysplasia accompanied by an abnormal growth plate morphology. We reveal that loss of *Stat3*, at least in part, downregulates Sox9 levels *in vivo* and results in a suite of phenotypic aberrations that appear to closely resemble many of those apparent in the *Sox9*^*+/-*^ haploinsufficient model [7]. Extensive *ex vivo* evaluation suggested that a measurable portion of the regulation of *Sox9* can likely be mediated directly by Stat3 binding to the *Sox9* promoter, at least in cell culture models. In total, our data suggest that the likely mechanism for the pathology involves disrupted Sox9 levels in the mutant mice and constitute, to our understanding, the first evidence of a non *Sox9*-associated genetic lesion resulting in a campomelic-like phenotype.

As Stat3 is thought to be a ubiquitous latent cytoplasmic protein, activated in a post-translational manner by phosphorylation, we initially hypothesized a rather broad domain of expression in developing embryos. However, our investigation into the developmental function of Stat3 following germ layer specification revealed a somewhat surprising likelihood of more pointed importance in tissues of mesodermal origin. Indeed, our mesodermal *Stat3* loss-of-function mutant animals developed dramatic perinatal skeletal abnormalities and quickly perished. Conditional loss-of-function analyses done prior to this study have suggested that Stat3 is required for the proper commitment and subsequent function of both osteoblasts and osteoclasts; however, these studies indicate a much less severe suite of skeletal issues due to defects in mineralization balance [31–33]. To our knowledge, the mice described in these studies do not exhibit a dwarf-like phenotype and generally survive into maturity with one minor exception being a reported incidence of small size accompanying a curvature of the spine and ~8 week survival in approximately 10 percent of mutant mice where *Stat3* was deleted from early osteoblasts and osteocytes [33]. The authors make no further analyses of these animals, and we suggest that this low-penetrant phenotype may arise from the reported sporadic *Col3*.*6Cre* activity within the chondrocyte pool [47]. Deletion of *Stat3* specifically from the chondrocyte population has not been reported; however, the growth plate architecture in mouse models where signaling, putatively through Stat3, is disrupted have failed to identify dramatic changes in chondrocyte biology [40, 41, 48]. Here, we suggest that early deletion of *Stat3* from the osteochondro progenitor pool by either *TCre* or *Prx1Cre* disrupts the dynamics of growth plate chondrocytes, indicating signaling independent of, or in concert with LIF is likely facilitating Stat3 function in these cells. Though we cannot rule out a distinct, Sox9-independent role for Stat3 within this growth plate defect, our interpretation leads us to believe this to be a major causative mechanism at work in our mutants.

With regard to how faithfully deletion of *Stat3* recapitulates the *Sox9* haploinsufficient model of CD, a majority of the pathologies described in the *Sox9*^*+/-*^ models are, to some extent, found in our array of Cre-driven mutant mice (Table 1) [7, 49]. Our panel of mutant mice suffer premature lethality, bending of bones, hip girdle dysplasia, hypoplastic tracheal cartilage, cleft palate and hypertrophic chondrocyte perdurance. We also demonstrate an additional feature, unreported in the *Sox9*^*+/-*^ model, i.e., spinal column curvature defects, which faithfully mirror the human clinical pathology of CD. Interestingly, the observation of spinal curvature and loss of intervertebral tissues was more recently described in an adult mouse model where *Sox9* was conditionally deleted at sexual maturity, bearing a striking similarity to our *TCre;Stat3*^*flox/Δ*^ mutant mice and lending support to our model [49]. Though we report a reduction in the physical and functional level of *Sox9* in our mutant mice, there is a delay in the onset of many of these features when compared to mice that are haploinsufficient for *Sox9*, suggesting that Stat3 may be necessary for fine-tuning *Sox9* expression within the developing growth plate. There, it may help maintain a critical threshold level for proper endochondral growth, with perhaps a greater requirement postnatally. In support of this idea, loss-of-function *Stat3* mutants have prominent deltoid tuberosities associated with their humeri (see Fig 3A and S3A and S3B Fig), a feature which is absent in the *Sox9*^*+/-*^ model, and may be explained either by Sox9-sensitive genetic background modifiers or potentially by lower over all levels of Sox9 in the haploinsufficient model [7]. One additional striking difference that our *Stat3* loss-of-function mutants display is the high incidence of spontaneous fractures. Besides endochondral ossification *per se*, loading forces experienced *in utero* by the surrounding skeletal muscle are known to exert mechanical stress on developing bones to properly shape them [50]. *Sox9*^*+/-*^ animals exhibit an osteosclerotic mineralization defect, which potentially resists this force; whereas, loss of *Stat3* results in brittle bones due to defects in osteoblast/osteoclast function, which cannot withstand these forces and may thus result in fractures [7]. Furthermore, while our *TCre;Stat3*^*flox/Δ*^ mutant mice deregulate *Sox9* expression only in the mesoderm and do not develop immediate respiratory dysfunction associated with CD, the high rate of perinatal lethality exhibited by our *Sox9Cre;Stat3*^*flox/Δ*^ mutant mice may be attributed to this additional insult.

The consequences of *Sox9* depletion are well known, but the mechanistic regulation of *Sox9 in vivo* is poorly understood. Recently, epigenetic control of the proximal *Sox9* promoter in a chondrogenesis culture model has been reported as a consequence of the balance between Wnt and fibroblast growth factor (Fgf) signaling, though physical activation of *Sox9* expression was not addressed [45]. It has also been reported that hypoxia inducible factor 1-α (HIF1α) can directly activate *Sox9* in culture and is required for appropriate development of skeletal elements of the autopod *in vivo* [51]. Additionally, Fgf signaling plays a well-established role in bone development and has been linked, in culture, with activation of *Sox9* expression mediated by ERK1/2 signaling [39]. As with our deletion of *Stat3*, chondrogenesis and bone development progress, albeit abnormally, in the aforementioned studies arguing for the involvement of multiple, cooperative signaling modalities in the regulation of *Sox9*. Of note, a recent study by Kondo et al., suggests that chondrogenic differentiation of human mesenchymal stem cells (MSCs) is mediated in part and subsequently enhanced by IL-6 signaling through STAT3, suggesting a likely conservation of the signaling events described in our present study [52]. Interestingly, several studies have previously indicated cooperative target gene activation by Stat3 and HIF1α, as well as the activation of Stat3 through direct phosphorylation by the Fgf receptor in response to Fgf signaling, suggesting that these pathways may converge and synergize on *Sox9* in the regulation of chondrogenesis [53–55].

The physical association of Stat3 with the *Sox9* proximal promoter is not entirely without precedent as other members of the Sox family, e.g., *Sox2* and *Sox6*, are activated directly by Stat3, implicating Stat3 signaling in multiple early developmental paradigms [56, 57]. While our cell culture experiments suggest Stat3’s association with the *Sox9* promoter, it is important to note that these data are merely suggestive of the mechanism *in vivo*. Further, it is possible that additional or more important binding elements exist within the much larger ~1Mb upstream regulatory regions of *Sox9*, and these questions warrant future investigation. Additionally, Stat-binding elements are heterogeneous and can be bound by multiple members of the Stat family [58]. In fact, Stat signaling in general is quite complex as multiple ligand and receptor interactions can stimulate the phosphorylation of multiple Stat family members either independently or coordinately (for review see [15]). It may be that redundancy within the Stat family can partially compensate for the loss of Stat3 to allow sufficient activation of *Sox9* early in the bone formation process. This compensation may also account for the seemingly normal skeletal development *in utero*, which is overcome early in the post-natal period and manifests as *Sox9* haploinsufficient-associated CD. Additionally, it is quite possible that the regulation of the *Sox9* locus by Stat3 may occur in tissues other than the skeletal system, and may therefore merit further attention in these contexts. Indeed, Sox9 has been shown to be involved in both the normal development and subsequent pathological calcification of cardiac valves and septa [59, 60]. Further, Sox9 is implicated in the proper morphogenesis of mammalian kidney and, in humans, is critical for proper gonad formation [2, 61]. Outside of normal development, and of wide potential impact, it has become a target of interest for its emerging role as an oncogene, suggesting that elucidation of key modulators, e.g., Stat3, could prove to be therapeutically important [62, 63].

From a clinical perspective, it is clear that the dominant genetic insult in CD is the mutation or deletion of the *SOX9* coding region. These lesions account for the preponderance of textbook CD with bowed limb elements [3–5]. CD can arise as milder phenotypes, to the point where no bending of the bones is evident, suggesting a heterogeneous pathology. To date, this phenomenon has largely been associated with chromosomal rearrangements outside of the *SOX9* coding region [8, 9, 64, 65]. We suggest that potential modifiers of *Sox9* expression, like Stat3 shown here, rather than frank mutations within the coding region may impart a portion of this milder heterogeneity. Additionally, there appears to be a minority subset of the disease where no detectable SOX9 mutations or rearrangements of known significance are described [5]. While Stat3 involvement is almost certainly not the sole explanation, it may be that in many cases of mild CD or ACD, the ability of Stat3 to access the promoter is limited or ablated. This could in turn cause the loss of sufficient levels of SOX9 and a muted version of CD and such a scenario warrants further investigation.

For frank CD, treatment is still largely based on ventilation at birth to circumvent breathing issues or on surgical interventions, as warranted for disambiguation of genitalia, hip dislocations, clubbed feet and spinal stabilization outside of the neonatal period. Though likely a complicated biological issue, it would be interesting to determine if amplification of Stat3 activation during gestation could rescue the effects of *Sox9* haploinsufficiency by upregulating the wild-type *Sox9* allele. Precedent for this type of treatment exists, in theory, as *in utero* administration of BMP2/7 rescues the skeletal defects associated with a mouse model of Rubinstein-Taybi syndrome [66]. Determination of the upstream ligands and receptors responsible for normal Stat3 activation within the early Sox9-expressing cells is necessary. As *LIF*^*-/-*^ and *LIFR*^*-/-*^ animals have normal growth plate architecture, it is likely that alternate cytokines and receptors for Stat3 activation, either alone or in conjunction with multiple components, are at work *in vivo* and as such, determination of these upstream events is currently part of our ongoing effort.

## Materials and methods

### Cell culture

NIH3T3 mouse fibroblasts (ATCC) were maintained in DMEM (Quality Biological) containing 10% FBS, 2 mM l-glutamine and 1% penicillin/streptomycin. For primary rat limb bud cell culture, fore and hind limb buds were carefully dissected from the body wall of F344 rat embryos at 13.5dpc and pooled. The buds were washed with PBS, briefly digested in 0.05% trypsin/0.53mM EDTA (Corning), gently washed with three exchanges of PBS and resuspended by trituration in basal media consisting of DMEM:F12 (Corning) supplemented as described with the addition of TGF-α (Peprotech), ROCK inhibitor Y27632 (Tocris), FGF2 (Peprotech) and LIF (Millipore) as indicated [67]. Where indicated, cells were treated with bovine serum albumin or dimethyl sulfoxide (both from Sigma) as controls for parallel treatment by IL-6, OSM (Cell Signaling Technology), LIF (EMD Millipore), or Stat3i (a gift from Nadya Tarasova, National Cancer Institute, Frederick, Maryland, USA).

### Plasmids and antibodies

Control luciferase plasmids, pGL3-basic and pRL-TK-renilla were from Promega. Sox9 luciferase reporter (pE2.4-Sox9Luc) was a gift from Peter Koopman (University of Queensland, Brisbane, Queensland, Australia). The pE770Sox9-Luc reporter was generated by *KpnI* digestion of pE2.4-Sox9Luc and vector re-ligation. Individual ΔSRE luciferase mutants were generated using the Quick Change II site-directed mutagenesis kit (Agilent Technologies) and the wild-type pE2.4-Sox9Luc template according to manufacturer’s protocol. The subsequent tandem ΔSRE constructs were generated by sequential rounds of site-directed mutagenesis using the appropriate single-site template constructs and all materials were sequence verified before use. The mutagenic primers were as follows: ΔSRE1 Fwd—GACATGCAATGCTAGGAACACCGGTTTGAAAAGAAACTTCGACTG, Rev—CAGTCGAAGTTTCTTTTCAAACCGGTGTTCCTAGCATTGCATGTC; ΔSRE2 Fwd—GTTTGAAAAGAAACCCGGACTGAACAGAGTTGTAGCTTGCTGC, Rev—GCAGCAAGCTACAACTCTGTTCAGTCCGGGTTTCTTTTCAAAC; ΔSRE3 Fwd—CCAAATAACAAATGCCCACCCGGGAAGAAAACGAGAGGAAAACG, Rev—CGTTTTCCTCTCGTTTTCTTCCCGGGTGGGCATTTGTTATTTGG. The following antibodies were used: anti-Sox9 (Millipore); anti-β-actin (Sigma); anti-Stat1, anti-Stat3, anti-phospho-Stat3 (Y705), anti-phospho-Stat1 (Y701), anti-phospho-Stat5 (Y694), and anti-phospho-ERK1/2 (all from Cell Signaling Technology); anti-Stat5A (Santa Cruz); alkaline phosphatase-conjugated anti-digoxigenin Fab (Roche); alexa-488-conjugated anti-rabbit IgG (Invitrogen); IR-680-conjugated anti-rabbit IgG and IR-800CW-conjugated anti-mouse IgG (both from Licor).

### Breeding of mice

*Stat3*^*flox/flox*^ mice (a gift from Shizuo Akira, Osaka University, Osaka, Japan) were intercrossed with a β-actinCre deleter strain (a gift from Mark Lewandoski, National Cancer Institute, Frederick, Maryland, USA) to generate germline deletion of a single allele of *Stat3* [68]. The resultant heterozygous (*Stat3*^*Δ/+*^) mice were then intercrossed with *TCre* (a gift from Mark Lewandoski), *Prx1Cre* or *Sox9Cre* (both gifts from Susan Mackem, National Cancer Institute, Frederick, Maryland, USA) to generate specific driver strains. These strains were then re-crossed with *Stat3*^*flox/flox*^ mice to generate tissue-specific deletion of *Stat3*. Female *R26R*^*LacZ*^ reporter mice (a gift from Mark Lewandoski) were crossed with male *TCre* drivers to perfom the indicated lineage trace. All mice were maintained on a C57BL/6 background and routinely genotyped by PCR (primer sequences available upon request).

### In situ hybridization (ISH)

Whole mount ISH was performed, essentially, as previously described [69] except BM purple (Roche) was used as the chromogenic substrate. Briefly, plasmids encoding cDNA for mouse *Stat3*, *Scx*, *MyoG*, or *Sox9* were linearized and DIG-labeled complimentary probes were generated using a DIG-labeling kit (Roche). As indicated, probes were hybridized overnight and detected using alkaline phosphatase-labeled anti-DIG antibody with subsequent chromogenic substrate development. For sectioning, embryos stained as above were embedded into paraffin wax after clearing in CitriSolv (Fisher Scientific) and sectioned serially at 16μM by microtome.

### Skeletal preparations

For gross skeletal analysis, skeletons were prepared essentially as described by McLeod [70]. Briefly, mice were euthanized according to institutional ACUC protocols, skinned, eviscerated, debulked of subcutaneous fat deposits and fixed in 95% ethanol. Skeletons were then treated in 100% acetone for 24-48hours (depending on age) and subsequently stained with Alcian Blue and Alizarin Red S. Finally, soft tissue digestion and specimen clearing was done in 1% potassium hydroxide. Preparations were stored and photographed in a 1:1 glycerol:ethanol solution using an Olympus SZX16 stereoscope and measurements were made by CellSens Dimension software.

### Histology and IHF

For histological analyses, paraffin sections of bones were produced from E18.5, P0 and P4-aged mice from the indicated genetic crosses. Limbs were dissected, skinned and fixed in 4% paraformaldehyde in PBS. Tissues were dehydrated through a graded series of ethanol, cleared in xylene, and embedded into paraffin for sectioning. To visualize the growth plates, sections were stained with Alcian Blue.

For immunolocalization by fluorescence, sections were dewaxed and rehydrated, and heat-mediated antigen retrieval was performed in citrate buffer (pH 6.0). Sections were blocked in 3% normal goat serum (NGS) containing 0.1% Triton X-100 for 1 hour at room temperature and subsequently incubated with antibody specific for the indicated target in 3% NGS overnight at 4°C. Sections were then washed in 1X Tris-buffered saline (TBS) and incubated with Alexafluor-488 conjugated secondary antibody in 3% NGS for 1 hour at room temperature. Sections were washed in TBS and countersained/mounted in vectashield containing DAPI (Vector Labs).

### Quantitative RT-PCR (qPCR)

Humeri from control and mutant animals were dissected, combined, and dounce-homogenized in 1ml Trizol reagent (Invitrogen) at the indicated age. For NIH3T3 cells, cells were grown to 50% confluence in 12-well plates and serum starved overnight. Cells then were treated as indicated and lysed in 500μl of Trizol reagent. Total RNA was recovered using an RNeasy Kit (Qiagen) and quantified by spectrophotometric analysis (NanoDrop). cDNA was reverse-transcribed from equal amounts of total RNA using random hexamer priming and the Verso cDNA Synthesis Kit (Thermo Scientific). qPCR was then performed on equal volumes of cDNA using a SsoFAST EvaGreen 2X PCR master mix (Biorad) in a CFX96 Real-Time System (BioRad). Data were normalized to GAPDH levels. Primers used were as follows: Sox9 Fwd, GAGGAAGTCGGTGAAGAACG, Sox9 Rev, CTGAGATTGCCCAGAGTGCT; Col2a1 Fwd, AAAGGGGCAGAAAGGAGAA, Col2a1 Rev, AGGATTTCCAGGGGTACCAG; GAPDH Fwd, AATGTGTCCGTCGTGGATCTG GAPDH Rev, CTGCTTCACCACCTTCTTGATGT.

### Transient Stat knockdown

Reverse transfection of indicated siRNAs was performed with RNAiMax (Invitrogen) according to manufacturer’s protocol in indicated media without antibiotics. Briefly, 60pmols of siRNA was incubated with 1μl RNAiMax for 20 minutes at room temperature. Rat limb bud cells were plated in 12-well human fibronectin-coated plates (BD Biosciences) at a density of 1.5x10^5^ cells/well after siRNA complex formation such that final total siRNA concentration was 50nM. The following siRNA constructs were used (Ambion/Thermo Fisher Scientific): Control #1 (catalog 4390843), Stat1 (ID s129044), Stat3-1 (ID s129046), Stat3-2 (ID s129047), Stat5A (s128672) and Stat5B (ID s129049).

### Immunoblotting

NIH3T3 or primary rat limb bud cells were treated as indicated and harvested for protein analysis by cell-lysis in SDS sample buffer (2% SDS, 10% glycerol and 60mM Tris-HCl, pH 7.5) followed by DNA hydrolysis at 100°C for 10 minutes. Total protein was quantified using the BioRad DC Protein Assay kit and equivalent amounts were subjected to SDS-PAGE on 4–12% Bis-Tris gels (Invitrogen). Gels were then transferred to nitrocellulose membranes, which were subsequently blocked in Odyssey diluent (Licor) and probed for indicated proteins with specific antibodies. Detection of protein-antibody complexes was carried out by IR dye-labeled secondary antibody and fluorescent capture using an Odyssey scanner (Licor). Color images were subsequently converted to greyscale for publication using Odyssey software (Licor). Where indicated, pixel intensities were captured using Odyssey software for quantitative analyses.

### Luciferase reporter assay

NIH3T3 cells were plated into 24-well plates and grown to 50% confluency. The indicated control or Sox9 (0.5μg) and *Renilla* (0.05μg) reporter constructs were co-transfected using Fugene 6 (Roche) in serum-free conditions. 1x10^6^ rat limb bud cells were nucleofected by Amaxa (Lonza) protocol DN-100 in P3 primary cell solution with control or Sox9 (2μg) and Renilla (0.2μg) reporter constructs and plated in indiciated media. 24 hours post-transfection, cells were treated as indicated in the absence of serum, for a further 24 hours and harvested by passive lysis. Luciferase activities were measured using the Dual-Luciferase Assay Kit (Promega) and a MicroLumat*Plus* luminometer (Berthold Technologies).

### Chromatin immunoprecipitation (ChIP)

ChIP was carried out essentially as described previously[71]. Briefly, NIH3T3 cells were grown to 75% confluence in 100-mm dishes and subsequently serum-starved overnight (16h). As indicated, cells were treated in the absence of serum and fixed in formaldehyde (1% in medium) for 15 minutes at room temperature. Nuclear extracts were prepared, and DNA was sheared by sonication in a Bioruptor (Diagenode) at 4°C to an average fragment size of 500bp as visualized by agarose gel. Equivalent volumes of nuclear extract were immunoprecipitated overnight with anti-Stat3 bound to protein G-sepharose beads (Sigma) at 4°C and eluted the following day by boiling in SDS elution buffer (50mM Tris-HCl, pH 8.0, 10mM EDTA, 1% SDS, 50mM NaHCO_3_). Crosslinking of “input” and immunoprecipitated samples was reversed in proteinase-K at 65°C overnight, and DNA was precipitated with phenol/chloroform by standard methods. DNA was analyzed by PCR using previously reported specific primers for *Socs3* [72] or the following regions of the *Sox9* promoter: SRE-Fwd, AGAAACTTCGACTGAACAGAGTTGT, SRE-Rev, AAGTGGGCATTTGTTATTTGG; Control-Fwd, TCGGCTTTGGTTTTCATTG, Control-Rev, AAATGTTTGGGTGACTCAACG.

### Immunofluorescence

NIH3T3 cells were grown on poly-l lysine (Sigma) coated coverglass until 50% confluence and subsequently serum starved overnight. Cells were treated, as indicated, for 30 minutes and immediately fixed in 2% paraformaldehyde at room temperature for 10 minutes. Coverslips were then blocked in 3% normal goat serum (NGS) containing 0.1% Triton X-100 for 1h at room temperature and incubated overnight in indicated primary antibodies. Samples were then washed in 1X Tris-buffered saline (TBS) and incubated with Alexafluor-488 and Alexafluor-568 conjugated secondary antibodies for 1 hour at room temperature. Coverglasses were washed in TBS and mounted in vectashield containing DAPI (Vector Labs).

### Statistics and probability analysis

Data were analyzed by Student’s t-test to ascertain the statistical significance of observations. Statistical differences were considered significant if p≤0.05* or p≤0.01**. All animal comparisons are made based on a minimum of three (n = 3) control and mutant littermates. All other experiments were performed a minimum of three independent times, and graphical data represents mean ± SEM (standard error of the mean). The probability of Stat consensus sequence presence was calculated using the following formula:

$$\text{Probability of site }=\;\left(1-\;\left[{\left(1-\text{p}\right)}^{\text{N}*\text{bp}}\right]\;*\;100\right)$$

where;

p = 1/(#nucleotides^k-mer^) = 1/(4^10^)

N = number of strands of DNA available for interrogation = 2

bp = length in bases of DNA interrogated = 2100

### Study approval

All animal procedures were performed following the guidelines from the NCI-Frederick Animal Care and Use Committee under an approved animal study proposal. NCI-Frederick is accredited by AAALAC International and follows the Public Health Service Policy for the Care and Use of Laboratory Animals. Animal care was provided in accordance with the procedures outlined in the "Guide for Care and Use of Laboratory Animals" (National Research Council; 1996; National Academy Press; Washington, D.C.).

## Supporting information

S1 Fig Characterization of background levels of WISH staining using a *Stat3* sense control probe in the mid-gestation mouse. (**A**) Lateral view of wild-type mouse embryo at E8.5 hybridized with sense probe. (**B**) Dorsal view of posterior region from A. (**C**) Lateral view of wild-type mouse embryo at E10.5 hybridized with sense probe. (**D**) Transverse section denoted in C (dashed line), NT—neural tube, Som—somites. (**E**) Higher magnification of box denoted in C, fl—forelimb. (**F**) Magnification of box shown in C. Outline demarcates apical ectodermal ridge (AER), fl—forelimb.
(TIF)

S2 Fig Late postnatal phenotype of *Stat3*-deficient mice. (**A**) Representative immunoblot of protein isolates from neonatal littermates demonstrating ablation of Stat3 in humeri. (**B**) Representative control (*TCre;Stat3*^*flox/+*^, left) and mutant (*TCre;Stat3*^*flox/Δ*^, right) ventral images of littermates at P14. Arrows indicate abnormal limb curvatures. Bar = 1cm.
(TIF)

S3 Fig Loss of Stat3 does not alter normal segmentation. (**A** and **B**) Expression analysis for *Myogenin* (*MyoG*) or *Scleraxis* (*Scx*) by whole mount in situ hybridization in control and *TCre;Stat3*^*flox/Δ*^ littermates at E12.5.
(TIF)

S4 Fig Abnormal skeletogenesis in *TCre;Stat3*^*flox/Δ*^ mice is progressive. (**A** and **B**) Alizarin Red/Alcian Blue-stained skeletal preps demonstrating bending and spontaneous fracture of forelimbs in *TCre;Stat3*^*flox/Δ*^ mice at indicated ages. Arrows indicate fractures of radius/ulna, dt—deltoid tuberosity. (**C, C’, D** and **D’**) Alizarin Red/Alcian Blue-stained skeletal preps depicting dysplastic hip girdles in *TCre;Stat3*^*flox/Δ*^ mice at specified ages. Arrows indicate sites of spontaneous fracture, brackets denote width of acetabular cartilage. (**E** and **F**) Alizarin Red/Alcian Blue-stained skeletal preps demonstrating antero-posterior compression of vertebral elements in *TCre;Stat3*^*flox/Δ*^ mice at indicated ages. Double-headed arrows indicate length of mutant vertebral body, L1—1^st^ lumbar vertebrae. (**G**) Box-and-whisker plots for radii, humeri, fibulae and femoral lengths (mm). Error bars represent SEM, n/s—not significant, *p<0.05, **p<0.01.
(TIF)

S5 Fig Analysis of tibial bowing. (**A**) Representative images and captured angular measurements of Alizarin Red/Alcian Blue-stained hindlimbs from P7 littermates. (**B**) Magnification of insets marked in A demonstrating representative points of measurement.
(TIF)

S6 Fig Stat3 is normally expressed in epiphyses. (**A**) Immunofluorescent analysis of Stat3 in serial sections from E14.5 control proximal humeri. (**B** and **C**) Representative depiction of distal humerus before and after epiphyseal dissection. (**D**) Representative immunoblot analysis of lysates generated from epiphyseal dissections from *TCre;Stat3*^*flox/+*^ *TCre;Stat3*^*flox/Δ*^ noted in C, at indicated ages.
(TIF)

S7 Fig Skeletal abnormalities manifested by *Prx1Cre*-mediated ablation of *Stat3*. (**A** and **B**) Representative images of littermate matched control and *Prx1Cre;Stat3*^*flox/Δ*^ mutant mice at P0 and P4. Arrows demonstrate bowing of forelimbs, bar = 1cm. (**C**) Chart depicting average weights of indicated genotypes in aging mice. Error bars are SEM, n/s—not significant, **p<0.01. (**D** and **E**) Alizarin Red/Alcian Blue-stained skeletal preps demonstrating bending and spontaneous fracture of forelimbs in *Prx1Cre;Stat3*^*flox/Δ*^ mice at indicated ages. Arrows indicate fractures of radius/ulna, asterisk denotes humerus, dt—deltoid tuberosity. (**F** and **G**) Alcian Blue-stained longitudinal sections of the proximal humeri of control and mutant *Prx1Cre;Stat3*^*flox/Δ*^ mice at indicated ages. Brackets denote the length of the hypertrophic chondrocyte region.
(TIF)

S8 Fig TCre effects recombination in anterior regions at E12.5. (**A-C**) Representative whole mount β-galactosidase staining of E12.5 embryo from *TCre* driver crossed with *Rosa*^*LacZ*^ carriers at indicated assay lengths. (**D**) Caudal aspect of transverse section from region denoted in panel B. (**E**) Representative negative control embryo demonstrating specificity of β-galactosidase protocol. Arrowheads indicate dorsal neural tube (nt) recombination. Arrows indicate likely cranial neural crest locations where *TCre* is active and overlap with observed reduction of *Sox9* mRNA in Fig 6A.
(TIF)

S9 Fig Regulation of Stats do not downregulate ERK signaling in rat limb bud cell cultures. (**A**) Representative image of E13.5 F344 rat embryo and limb buds. Dotted lines indicate point of dissection for culture material. (**B**) Representative image of monolayer generated 8h post-dissection and culture. (**C**) Representative immunoblot demonstrating early induction of Stat3 activation in response to indicated stimuli in rat limb bud cells. F—Fgf2 (50ng/ml), T—TGFα (10ng/ml), Y—Y27632 (10μM). (**D**) Representative immunoblot demonstrating presence of indicated proteins in response to indicated siRNA treatments. (**E**) Quantitation of pERK1/2 levels in D. Error bars are SEM, *p<0.05, **p<0.01.
(TIF)

S10 Fig Species conservation of Stat binding in the *Sox9* promoter. (**A**) Schematic of immediate upstream *Sox9* homolog loci across indicated species. Red blocks indicate putative Stat DNA binding elements.
(TIF)

S11 Fig Response of cultured rodent cells to Stat activation. (**A**) Immunofluorescent analysis of Sox9 and Stat3 protein localization in NIH3T3 cells in response to stimulation by OSM for 30 minutes. (**B**) Total RNA isolated from control or IL-6 treated NIH3T3 cells analyzed for *Sox9* expression by quantitative RT-PCR after 30 minutes. Error bars represent SEM, *p<0.05. (**C** and **D**) Immunoblot analysis depicting activation of indicated proteins in response to IL-6 or OSM treatment at specified doses. Arrowheads indicate doublet isoforms of ERK. (**E** and **F**) Analysis of a *Sox9* promoter-driven luciferase construct in response to indicated treatment for 24h in rat limb bud cells grown in basal TY media (E) or FTLY media (F). Error bars are SEM. (**G**) Fold change analysis of a *Sox9* promoter-driven luciferase construct lacking the Stat-binding regions in response to control or OSM treatment in the presence of a Stat3 inhibitor for 24h. Error bars are SEM, n/s—not significant.
(TIF)

S1 Table Breeding scheme and expected genetic representation of offspring. *TCre* and *Prx1Cre* driver males were homozygous for the *Cre* transgene, where *Sox9Cre* males were heterozygous. Expected and observed offspring ratios at birth are indicated.
(TIF)

## References

[1] Maroteaux P, Spranger J, Opitz JM, Kucera J, Lowry RB, Schimke RN, et al [The campomelic syndrome]. Presse Med. 1971;79(25):1157–62. Epub 1971/05/22. 5555980

[2] Mansour S, Hall CM, Pembrey ME, Young ID. A clinical and genetic study of campomelic dysplasia. J Med Genet. 1995;32(6):415–20. Epub 1995/06/01. 7666392 10.1136/jmg.32.6.415 PMC1050480

[3] Foster JW, Dominguez-Steglich MA, Guioli S, Kwok C, Weller PA, Stevanovic M, et al Campomelic dysplasia and autosomal sex reversal caused by mutations in an SRY-related gene. Nature. 1994;372(6506):525–30. Epub 1994/12/08. 10.1038/372525a0 7990924

[4] Kwok C, Weller PA, Guioli S, Foster JW, Mansour S, Zuffardi O, et al Mutations in SOX9, the gene responsible for Campomelic dysplasia and autosomal sex reversal. Am J Hum Genet. 1995;57(5):1028–36. Epub 1995/11/01. 7485151 PMC1801368

[5] Meyer J, Sudbeck P, Held M, Wagner T, Schmitz ML, Bricarelli FD, et al Mutational analysis of the SOX9 gene in campomelic dysplasia and autosomal sex reversal: lack of genotype/phenotype correlations. Hum Mol Genet. 1997;6(1):91–8. Epub 1997/01/01. 9002675 10.1093/hmg/6.1.91

[6] Long F, Ornitz DM. Development of the endochondral skeleton. Cold Spring Harb Perspect Biol. 2013;5(1):a008334 Epub 2013/01/04. 10.1101/cshperspect.a008334 23284041 PMC3579395

[7] Bi W, Huang W, Whitworth DJ, Deng JM, Zhang Z, Behringer RR, et al Haploinsufficiency of Sox9 results in defective cartilage primordia and premature skeletal mineralization. Proc Natl Acad Sci U S A. 2001;98(12):6698–703. Epub 2001/05/24. 10.1073/pnas.111092198 11371614 PMC34415

[8] Pfeifer D, Kist R, Dewar K, Devon K, Lander ES, Birren B, et al Campomelic dysplasia translocation breakpoints are scattered over 1 Mb proximal to SOX9: evidence for an extended control region. Am J Hum Genet. 1999;65(1):111–24. Epub 1999/06/12. 10.1086/302455 10364523 PMC1378081

[9] Gordon CT, Tan TY, Benko S, Fitzpatrick D, Lyonnet S, Farlie PG. Long-range regulation at the SOX9 locus in development and disease. J Med Genet. 2009;46(10):649–56. 10.1136/jmg.2009.068361 19473998

[10] Wagner T, Wirth J, Meyer J, Zabel B, Held M, Zimmer J, et al Autosomal sex reversal and campomelic dysplasia are caused by mutations in and around the SRY-related gene SOX9. Cell. 1994;79(6):1111–20. 8001137 10.1016/0092-8674(94)90041-8

[11] Bagheri-Fam S, Barrionuevo F, Dohrmann U, Gunther T, Schule R, Kemler R, et al Long-range upstream and downstream enhancers control distinct subsets of the complex spatiotemporal Sox9 expression pattern. Dev Biol. 2006;291(2):382–97. 10.1016/j.ydbio.2005.11.013 16458883

[12] Mead TJ, Wang Q, Bhattaram P, Dy P, Afelik S, Jensen J, et al A far-upstream (-70 kb) enhancer mediates Sox9 auto-regulation in somatic tissues during development and adult regeneration. Nucleic Acids Res. 2013;41(8):4459–69. 10.1093/nar/gkt140 23449223 PMC3632127

[13] Wunderle VM, Critcher R, Hastie N, Goodfellow PN, Schedl A. Deletion of long-range regulatory elements upstream of SOX9 causes campomelic dysplasia. Proc Natl Acad Sci U S A. 1998;95(18):10649–54. Epub 1998/09/02. 9724758 10.1073/pnas.95.18.10649 PMC27949

[14] Yao B, Wang Q, Liu CF, Bhattaram P, Li W, Mead TJ, et al The SOX9 upstream region prone to chromosomal aberrations causing campomelic dysplasia contains multiple cartilage enhancers. Nucleic Acids Res. 2015;43(11):5394–408. 10.1093/nar/gkv426 25940622 PMC4477657

[15] Schindler C, Levy DE, Decker T. JAK-STAT signaling: from interferons to cytokines. J Biol Chem. 2007;282(28):20059–63. Epub 2007/05/16. 10.1074/jbc.R700016200 17502367

[16] Meraz MA, White JM, Sheehan KC, Bach EA, Rodig SJ, Dighe AS, et al Targeted disruption of the Stat1 gene in mice reveals unexpected physiologic specificity in the JAK-STAT signaling pathway. Cell. 1996;84(3):431–42. Epub 1996/02/09. 8608597 10.1016/s0092-8674(00)81288-x

[17] Durbin JE, Hackenmiller R, Simon MC, Levy DE. Targeted disruption of the mouse Stat1 gene results in compromised innate immunity to viral disease. Cell. 1996;84(3):443–50. Epub 1996/02/09. 8608598 10.1016/s0092-8674(00)81289-1

[18] Park C, Li S, Cha E, Schindler C. Immune response in Stat2 knockout mice. Immunity. 2000;13(6):795–804. Epub 2001/02/13. 11163195 10.1016/s1074-7613(00)00077-7

[19] Thierfelder WE, van Deursen JM, Yamamoto K, Tripp RA, Sarawar SR, Carson RT, et al Requirement for Stat4 in interleukin-12-mediated responses of natural killer and T cells. Nature. 1996;382(6587):171–4. Epub 1996/07/11. 10.1038/382171a0 8700208

[20] Kaplan MH, Sun YL, Hoey T, Grusby MJ. Impaired IL-12 responses and enhanced development of Th2 cells in Stat4-deficient mice. Nature. 1996;382(6587):174–7. Epub 1996/07/11. 10.1038/382174a0 8700209

[21] Takeda K, Tanaka T, Shi W, Matsumoto M, Minami M, Kashiwamura S, et al Essential role of Stat6 in IL-4 signalling. Nature. 1996;380(6575):627–30. Epub 1996/04/18. 10.1038/380627a0 8602263

[22] Shimoda K, van Deursen J, Sangster MY, Sarawar SR, Carson RT, Tripp RA, et al Lack of IL-4-induced Th2 response and IgE class switching in mice with disrupted Stat6 gene. Nature. 1996;380(6575):630–3. Epub 1996/04/18. 10.1038/380630a0 8602264

[23] Liu X, Robinson GW, Wagner KU, Garrett L, Wynshaw-Boris A, Hennighausen L. Stat5a is mandatory for adult mammary gland development and lactogenesis. Genes Dev. 1997;11(2):179–86. Epub 1997/01/15. 9009201 10.1101/gad.11.2.179

[24] Udy GB, Towers RP, Snell RG, Wilkins RJ, Park SH, Ram PA, et al Requirement of STAT5b for sexual dimorphism of body growth rates and liver gene expression. Proc Natl Acad Sci U S A. 1997;94(14):7239–44. Epub 1997/07/08. 9207075 10.1073/pnas.94.14.7239 PMC23803

[25] Teglund S, McKay C, Schuetz E, van Deursen JM, Stravopodis D, Wang D, et al Stat5a and Stat5b proteins have essential and nonessential, or redundant, roles in cytokine responses. Cell. 1998;93(5):841–50. Epub 1998/06/18. 9630227 10.1016/s0092-8674(00)81444-0

[26] Takeda K, Noguchi K, Shi W, Tanaka T, Matsumoto M, Yoshida N, et al Targeted disruption of the mouse Stat3 gene leads to early embryonic lethality. Proc Natl Acad Sci U S A. 1997;94(8):3801–4. Epub 1997/04/15. 9108058 10.1073/pnas.94.8.3801 PMC20521

[27] Yoshida K, Taga T, Saito M, Suematsu S, Kumanogoh A, Tanaka T, et al Targeted disruption of gp130, a common signal transducer for the interleukin 6 family of cytokines, leads to myocardial and hematological disorders. Proc Natl Acad Sci U S A. 1996;93(1):407–11. Epub 1996/01/09. 8552649 10.1073/pnas.93.1.407 PMC40247

[28] Oates AC, Wollberg P, Pratt SJ, Paw BH, Johnson SL, Ho RK, et al Zebrafish stat3 is expressed in restricted tissues during embryogenesis and stat1 rescues cytokine signaling in a STAT1-deficient human cell line. Dev Dyn. 1999;215(4):352–70. 10.1002/(SICI)1097-0177(199908)215:4<352::AID-AJA7>3.0.CO;2-J 10417824

[29] Takeda K, Kaisho T, Yoshida N, Takeda J, Kishimoto T, Akira S. Stat3 activation is responsible for IL-6-dependent T cell proliferation through preventing apoptosis: generation and characterization of T cell-specific Stat3-deficient mice. J Immunol. 1998;161(9):4652–60. Epub 1998/10/30. 9794394

[30] Perantoni AO, Timofeeva O, Naillat F, Richman C, Pajni-Underwood S, Wilson C, et al Inactivation of FGF8 in early mesoderm reveals an essential role in kidney development. Development. 2005;132(17):3859–71. Epub 2005/07/29. 10.1242/dev.01945 16049111

[31] Itoh S, Udagawa N, Takahashi N, Yoshitake F, Narita H, Ebisu S, et al A critical role for interleukin-6 family-mediated Stat3 activation in osteoblast differentiation and bone formation. Bone. 2006;39(3):505–12. Epub 2006/05/09. 10.1016/j.bone.2006.02.074 16679075

[32] Zhang Z, Welte T, Troiano N, Maher SE, Fu XY, Bothwell AL. Osteoporosis with increased osteoclastogenesis in hematopoietic cell-specific STAT3-deficient mice. Biochem Biophys Res Commun. 2005;328(3):800–7. Epub 2005/02/08. 10.1016/j.bbrc.2005.01.019 15694417

[33] Zhou H, Newnum AB, Martin JR, Li P, Nelson MT, Moh A, et al Osteoblast/osteocyte-specific inactivation of Stat3 decreases load-driven bone formation and accumulates reactive oxygen species. Bone. 2011;49(3):404–11. Epub 2011/05/11. 10.1016/j.bone.2011.04.020 21555004

[34] Suemoto H, Muragaki Y, Nishioka K, Sato M, Ooshima A, Itoh S, et al Trps1 regulates proliferation and apoptosis of chondrocytes through Stat3 signaling. Dev Biol. 2007;312(2):572–81. 10.1016/j.ydbio.2007.10.001 17997399

[35] Logan M, Martin JF, Nagy A, Lobe C, Olson EN, Tabin CJ. Expression of Cre Recombinase in the developing mouse limb bud driven by a Prxl enhancer. Genesis. 2002;33(2):77–80. Epub 2002/07/12. 10.1002/gene.10092 12112875

[36] Udagawa N, Takahashi N, Akatsu T, Tanaka H, Sasaki T, Nishihara T, et al Origin of osteoclasts: mature monocytes and macrophages are capable of differentiating into osteoclasts under a suitable microenvironment prepared by bone marrow-derived stromal cells. Proc Natl Acad Sci U S A. 1990;87(18):7260–4. Epub 1990/09/01. 2169622 10.1073/pnas.87.18.7260 PMC54723

[37] Akiyama H, Kim JE, Nakashima K, Balmes G, Iwai N, Deng JM, et al Osteo-chondroprogenitor cells are derived from Sox9 expressing precursors. Proc Natl Acad Sci U S A. 2005;102(41):14665–70. Epub 2005/10/06. 10.1073/pnas.0504750102 16203988 PMC1239942

[38] Soriano P. Generalized lacZ expression with the ROSA26 Cre reporter strain. Nat Genet. 1999;21(1):70–1. 10.1038/5007 9916792

[39] Murakami S, Kan M, McKeehan WL, de Crombrugghe B. Up-regulation of the chondrogenic Sox9 gene by fibroblast growth factors is mediated by the mitogen-activated protein kinase pathway. Proc Natl Acad Sci U S A. 2000;97(3):1113–8. Epub 2000/02/03. 10655493 10.1073/pnas.97.3.1113 PMC15539

[40] Poulton IJ, McGregor NE, Pompolo S, Walker EC, Sims NA. Contrasting roles of leukemia inhibitory factor in murine bone development and remodeling involve region-specific changes in vascularization. J Bone Miner Res. 2012;27(3):586–95. Epub 2011/12/07. 10.1002/jbmr.1485 22143976

[41] Sims NA, Johnson RW. Leukemia inhibitory factor: a paracrine mediator of bone metabolism. Growth Factors. 2012;30(2):76–87. Epub 2012/02/07. 10.3109/08977194.2012.656760 22304408

[42] Li G, Peng H, Corsi K, Usas A, Olshanski A, Huard J. Differential effect of BMP4 on NIH/3T3 and C2C12 cells: implications for endochondral bone formation. J Bone Miner Res. 2005;20(9):1611–23. 10.1359/JBMR.050513 16059633

[43] Kanai Y, Koopman P. Structural and functional characterization of the mouse Sox9 promoter: implications for campomelic dysplasia. Hum Mol Genet. 1999;8(4):691–6. Epub 1999/03/11. 10072439 10.1093/hmg/8.4.691

[44] Timofeeva OA, Gaponenko V, Lockett SJ, Tarasov SG, Jiang S, Michejda CJ, et al Rationally designed inhibitors identify STAT3 N-domain as a promising anticancer drug target. ACS Chem Biol. 2007;2(12):799–809. Epub 2007/12/25. 10.1021/cb700186x 18154267

[45] Kumar D, Lassar AB. Fibroblast growth factor maintains chondrogenic potential of limb bud mesenchymal cells by modulating DNMT3A recruitment. Cell Rep. 2014;8(5):1419–31. Epub 2014/08/28. 10.1016/j.celrep.2014.07.038 25159139 PMC4163101

[46] Auernhammer CJ, Bousquet C, Melmed S. Autoregulation of pituitary corticotroph SOCS-3 expression: characterization of the murine SOCS-3 promoter. Proc Natl Acad Sci U S A. 1999;96(12):6964–9. Epub 1999/06/09. 10359822 10.1073/pnas.96.12.6964 PMC22025

[47] Liu F, Woitge HW, Braut A, Kronenberg MS, Lichtler AC, Mina M, et al Expression and activity of osteoblast-targeted Cre recombinase transgenes in murine skeletal tissues. Int J Dev Biol. 2004;48(7):645–53. Epub 2004/10/08. 10.1387/ijdb.041816fl 15470637

[48] Sims NA, Jenkins BJ, Quinn JM, Nakamura A, Glatt M, Gillespie MT, et al Glycoprotein 130 regulates bone turnover and bone size by distinct downstream signaling pathways. J Clin Invest. 2004;113(3):379–89. Epub 2004/02/03. 10.1172/JCI19872 14755335 PMC324544

[49] Henry SP, Liang S, Akdemir KC, de Crombrugghe B. The postnatal role of Sox9 in cartilage. J Bone Miner Res. 2012;27(12):2511–25. Epub 2012/07/11. 10.1002/jbmr.1696 22777888 PMC3502666

[50] Sharir A, Stern T, Rot C, Shahar R, Zelzer E. Muscle force regulates bone shaping for optimal load-bearing capacity during embryogenesis. Development. 2011;138(15):3247–59. Epub 2011/07/14. 10.1242/dev.063768 21750035

[51] Amarilio R, Viukov SV, Sharir A, Eshkar-Oren I, Johnson RS, Zelzer E. HIF1alpha regulation of Sox9 is necessary to maintain differentiation of hypoxic prechondrogenic cells during early skeletogenesis. Development. 2007;134(21):3917–28. Epub 2007/10/05. 10.1242/dev.008441 17913788

[52] Kondo M, Yamaoka K, Sakata K, Sonomoto K, Lin L, Nakano K, et al Contribution of the Interleukin-6/STAT-3 Signaling Pathway to Chondrogenic Differentiation of Human Mesenchymal Stem Cells. Arthritis Rheumatol. 2015;67(5):1250–60. 10.1002/art.39036 25604648

[53] Pawlus MR, Wang L, Hu CJ. STAT3 and HIF1alpha cooperatively activate HIF1 target genes in MDA-MB-231 and RCC4 cells. Oncogene. 2014;33(13):1670–9. Epub 2013/04/23. 10.1038/onc.2013.115 23604114 PMC3868635

[54] Pawlus MR, Wang L, Murakami A, Dai G, Hu CJ. STAT3 or USF2 contributes to HIF target gene specificity. PLoS One. 2013;8(8):e72358 Epub 2013/08/31. 10.1371/journal.pone.0072358 23991099 PMC3749168

[55] Dudka AA, Sweet SM, Heath JK. Signal transducers and activators of transcription-3 binding to the fibroblast growth factor receptor is activated by receptor amplification. Cancer Res. 2010;70(8):3391–401. Epub 2010/04/15. 10.1158/0008-5472.CAN-09-3033 20388777 PMC2887080

[56] Snyder M, Huang XY, Zhang JJ. Stat3 is essential for neuronal differentiation through direct transcriptional regulation of the Sox6 gene. FEBS Lett. 2011;585(1):148–52. Epub 2010/11/26. 10.1016/j.febslet.2010.11.030 21094641 PMC3022371

[57] Foshay KM, Gallicano GI. Regulation of Sox2 by STAT3 initiates commitment to the neural precursor cell fate. Stem Cells Dev. 2008;17(2):269–78. Epub 2008/05/02. 10.1089/scd.2007.0098 18447642

[58] Ehret GB, Reichenbach P, Schindler U, Horvath CM, Fritz S, Nabholz M, et al DNA binding specificity of different STAT proteins. Comparison of in vitro specificity with natural target sites. J Biol Chem. 2001;276(9):6675–88. Epub 2000/10/29. 10.1074/jbc.M001748200 11053426

[59] Peacock JD, Levay AK, Gillaspie DB, Tao G, Lincoln J. Reduced sox9 function promotes heart valve calcification phenotypes in vivo. Circ Res. 2010;106(4):712–9. Epub 2010/01/09. 10.1161/CIRCRESAHA.109.213702 20056916 PMC2863131

[60] Akiyama H, Chaboissier MC, Behringer RR, Rowitch DH, Schedl A, Epstein JA, et al Essential role of Sox9 in the pathway that controls formation of cardiac valves and septa. Proc Natl Acad Sci U S A. 2004;101(17):6502–7. Epub 2004/04/21. 10.1073/pnas.0401711101 15096597 PMC404074

[61] Reginensi A, Clarkson M, Neirijnck Y, Lu B, Ohyama T, Groves AK, et al SOX9 controls epithelial branching by activating RET effector genes during kidney development. Hum Mol Genet. 2011;20(6):1143–53. Epub 2011/01/08. 10.1093/hmg/ddq558 21212101 PMC3809456

[62] Kopp JL, von Figura G, Mayes E, Liu FF, Dubois CL, Morris JPt, et al Identification of Sox9-dependent acinar-to-ductal reprogramming as the principal mechanism for initiation of pancreatic ductal adenocarcinoma. Cancer Cell. 2012;22(6):737–50. Epub 2012/12/04. 10.1016/j.ccr.2012.10.025 23201164 PMC3568632

[63] Matheu A, Collado M, Wise C, Manterola L, Cekaite L, Tye AJ, et al Oncogenicity of the developmental transcription factor Sox9. Cancer Res. 2012;72(5):1301–15. Epub 2012/01/17. 10.1158/0008-5472.CAN-11-3660 22246670 PMC3378515

[64] Ninomiya S, Isomura M, Narahara K, Seino Y, Nakamura Y. Isolation of a testis-specific cDNA on chromosome 17q from a region adjacent to the breakpoint of t(12;17) observed in a patient with acampomelic campomelic dysplasia and sex reversal. Hum Mol Genet. 1996;5(1):69–72. Epub 1996/01/01. 8789441 10.1093/hmg/5.1.69

[65] Leipoldt M, Erdel M, Bien-Willner GA, Smyk M, Theurl M, Yatsenko SA, et al Two novel translocation breakpoints upstream of SOX9 define borders of the proximal and distal breakpoint cluster region in campomelic dysplasia. Clin Genet. 2007;71(1):67–75. Epub 2007/01/06. 10.1111/j.1399-0004.2007.00736.x 17204049

[66] Shim JH, Greenblatt MB, Singh A, Brady N, Hu D, Drapp R, et al Administration of BMP2/7 in utero partially reverses Rubinstein-Taybi syndrome-like skeletal defects induced by Pdk1 or Cbp mutations in mice. J Clin Invest. 2012;122(1):91–106. Epub 2011/12/03. 10.1172/JCI59466 22133875 PMC3248303

[67] Tanigawa S, Sharma N, Hall MD, Nishinakamura R, Perantoni AO. Preferential Propagation of Competent SIX2+ Nephronic Progenitors by LIF/ROCKi Treatment of the Metanephric Mesenchyme. Stem Cell Reports. 2015;5(3):435–47. 10.1016/j.stemcr.2015.07.015 26321142 PMC4618653

[68] Lewandoski M, Meyers EN, Martin GR. Analysis of Fgf8 gene function in vertebrate development. Cold Spring Harb Symp Quant Biol. 1997;62:159–68. Epub 1997/01/01. 9598348

[69] Wilkinson DG, Green J. In situ hybridization and the three-dimensional reconstruction of serial section. Copp A.J. CDL, editor: Oxford: IRL Press; 1990.

[70] McLeod MJ. Differential staining of cartilage and bone in whole mouse fetuses by alcian blue and alizarin red S. Teratology. 1980;22(3):299–301. Epub 1980/12/01. 10.1002/tera.1420220306 6165088

[71] Lee TI, Johnstone SE, Young RA. Chromatin immunoprecipitation and microarray-based analysis of protein location. Nat Protoc. 2006;1(2):729–48. Epub 2007/04/05. 10.1038/nprot.2006.98 17406303 PMC3004291

[72] Hutchins AP, Diez D, Takahashi Y, Ahmad S, Jauch R, Tremblay ML, et al Distinct transcriptional regulatory modules underlie STAT3's cell type-independent and cell type-specific functions. Nucleic Acids Res. 2013;41(4):2155–70. Epub 2013/01/09. 10.1093/nar/gks1300 23295670 PMC3575808

