# Supplementary figures and images for: Mesoderm-specific Stat3 deletion affects expression of Sox9 yielding Sox9-dependent phenotypes
Source: PLoS Genet. 2017 Feb 6;13(2):e1006610. doi: 10.1371/journal.pgen.1006610 (PMC5319801; doi:10.1371/journal.pgen.1006610)

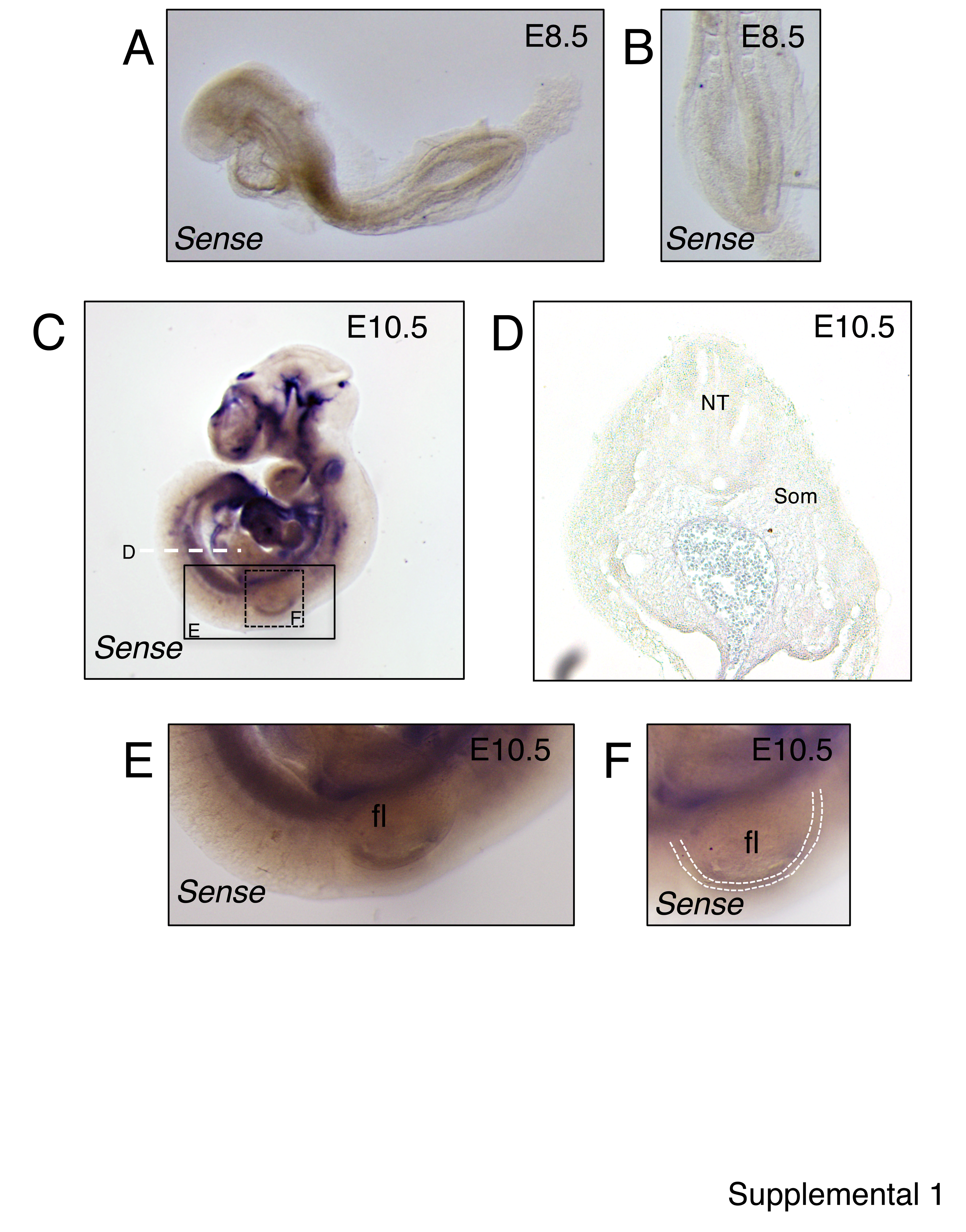

Supplement: S1 Fig — (A) Lateral view of wild-type mouse embryo at E8.5 hybridized with sense probe. (B) Dorsal view of posterior region from A. (C) Lateral view of wild-type mouse embryo at E10.5 hybridized with sense probe. (D) Transverse section denoted in C (dashed line), NT—neural tube, Som—somites. (E) Higher magnification of box denoted in C, fl—forelimb. (F) Magnification of box shown in C. Outline demarcates apical ectodermal ridge (AER), fl—forelimb. (TIF) [file pgen.1006610.s001.tif]

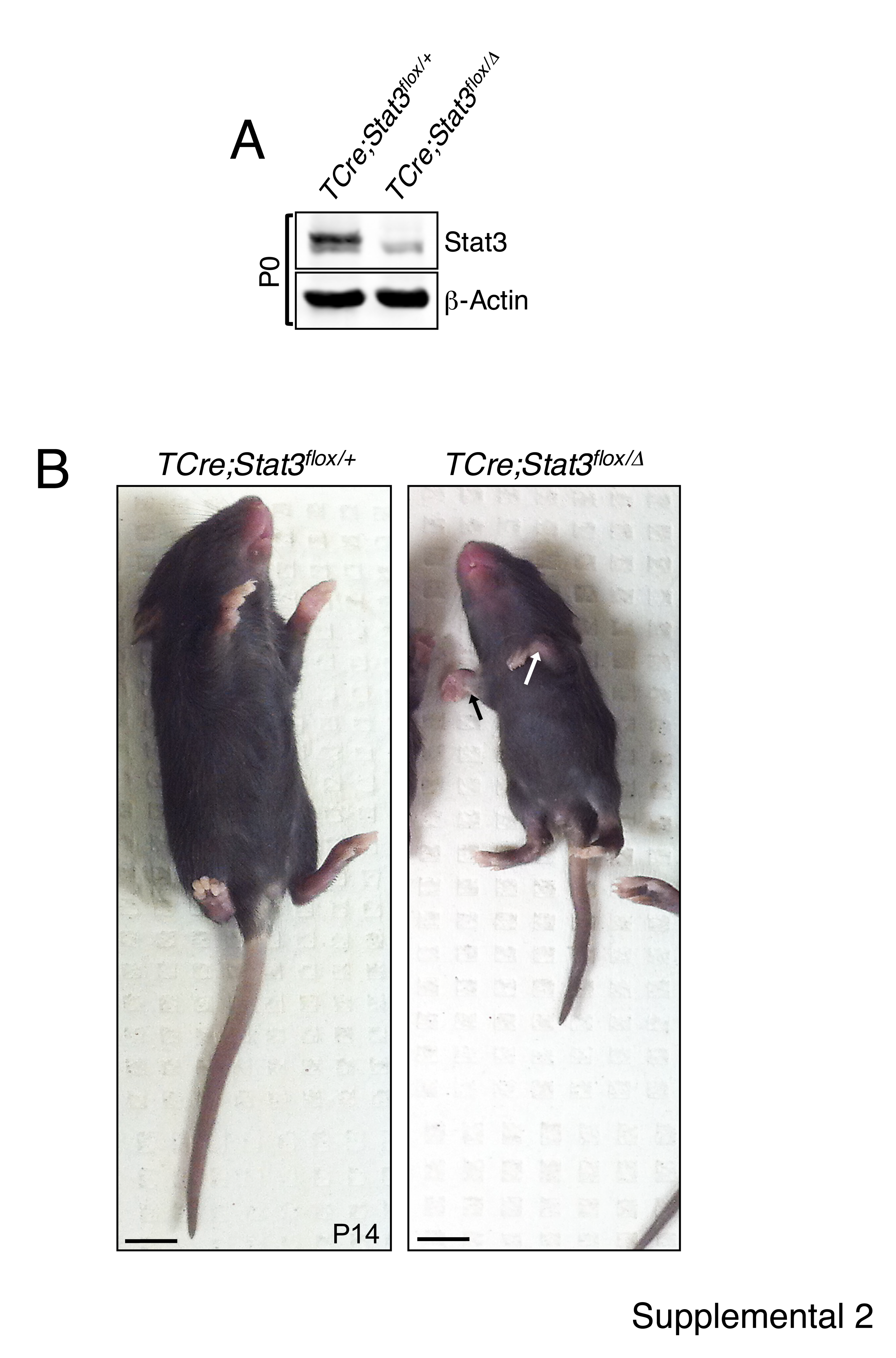

Supplement: S2 Fig — (A) Representative immunoblot of protein isolates from neonatal littermates demonstrating ablation of Stat3 in humeri. (B) Representative control (TCre;Stat3flox/+, left) and mutant (TCre;Stat3flox/Δ, right) ventral images of littermates at P14. Arrows indicate abnormal limb curvatures. Bar = 1cm. (TIF) [file pgen.1006610.s002.tif]

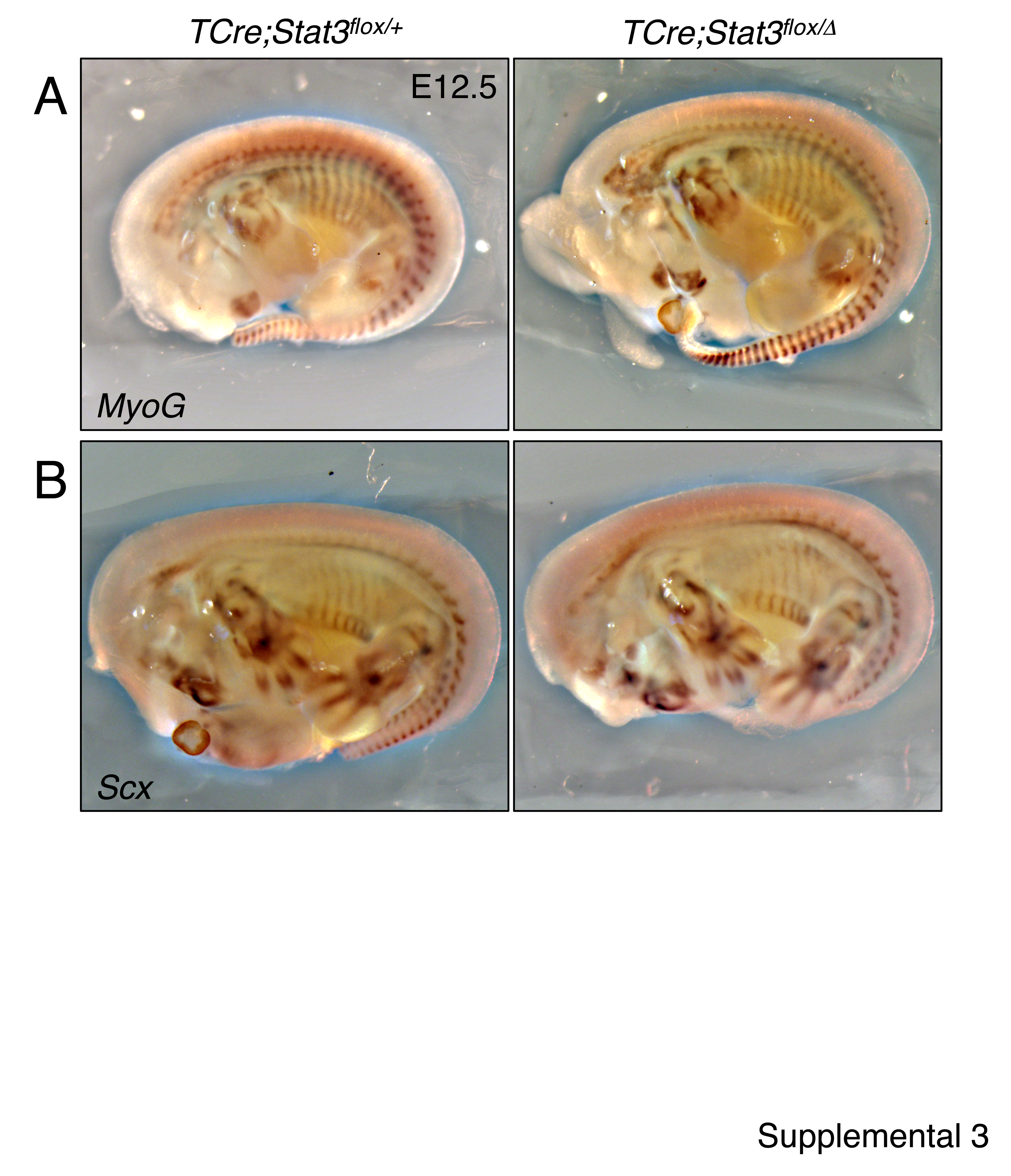

Supplement: S3 Fig — (A and B) Expression analysis for Myogenin (MyoG) or Scleraxis (Scx) by whole mount in situ hybridization in control and TCre;Stat3flox/Δ littermates at E12.5. (TIF) [file pgen.1006610.s003.tif]

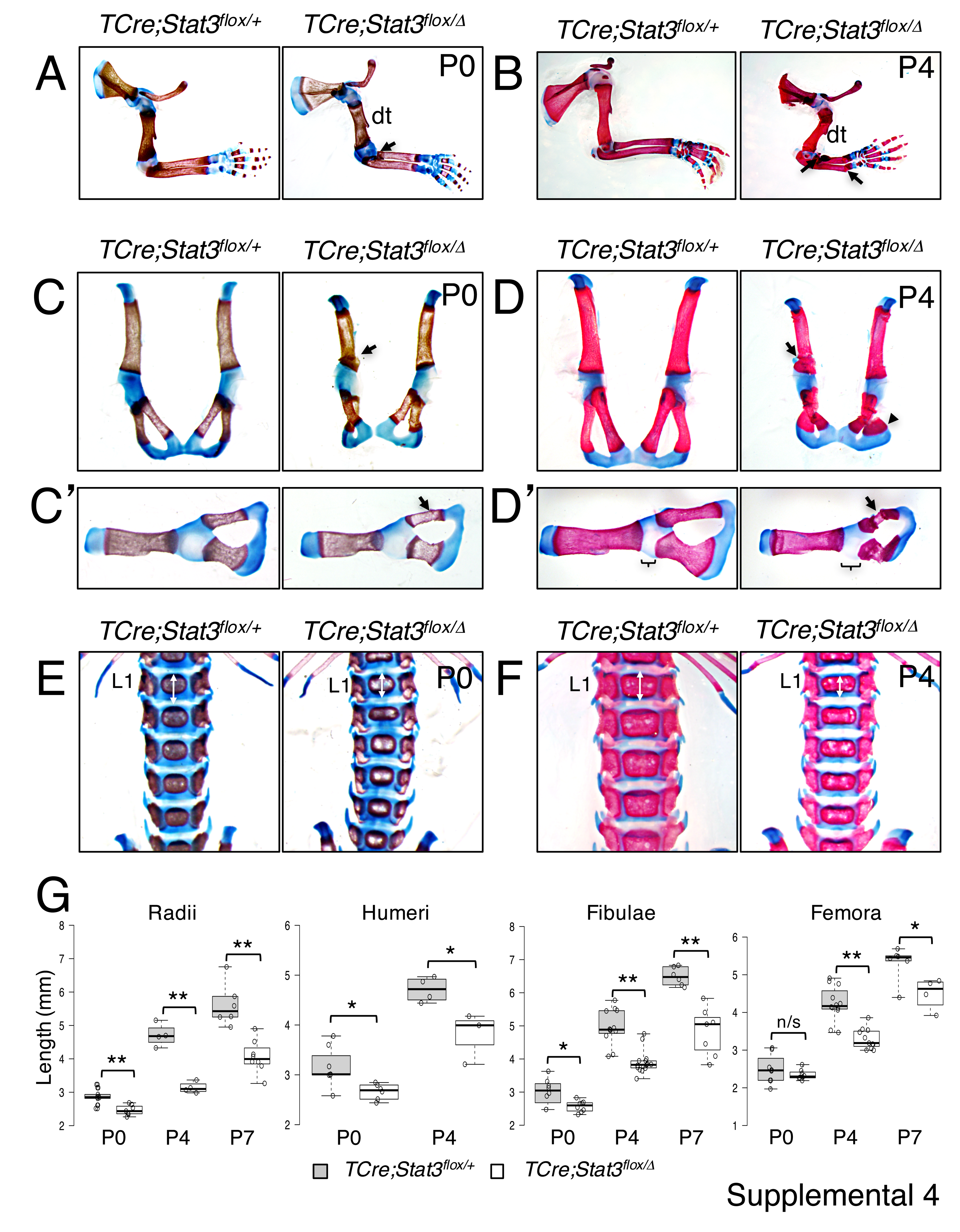

Supplement: S4 Fig — (A and B) Alizarin Red/Alcian Blue-stained skeletal preps demonstrating bending and spontaneous fracture of forelimbs in TCre;Stat3flox/Δ mice at indicated ages. Arrows indicate fractures of radius/ulna, dt—deltoid tuberosity. (C, C’, D and D’) Alizarin Red/Alcian Blue-stained skeletal preps depicting dysplastic hip girdles in TCre;Stat3flox/Δ mice at specified ages. Arrows indicate sites of spontaneous fracture, brackets denote width of acetabular cartilage. (E and F) Alizarin Red/Alcian Blue-stained skeletal preps demonstrating antero-posterior compression of vertebral elements in TCre;Stat3flox/Δ mice at indicated ages. Double-headed arrows indicate length of mutant vertebral body, L1—1st lumbar vertebrae. (G) Box-and-whisker plots for radii, humeri, fibulae and femoral lengths (mm). Error bars represent SEM, n/s—not significant, *p<0.05, **p<0.01. (TIF) [file pgen.1006610.s004.tif]

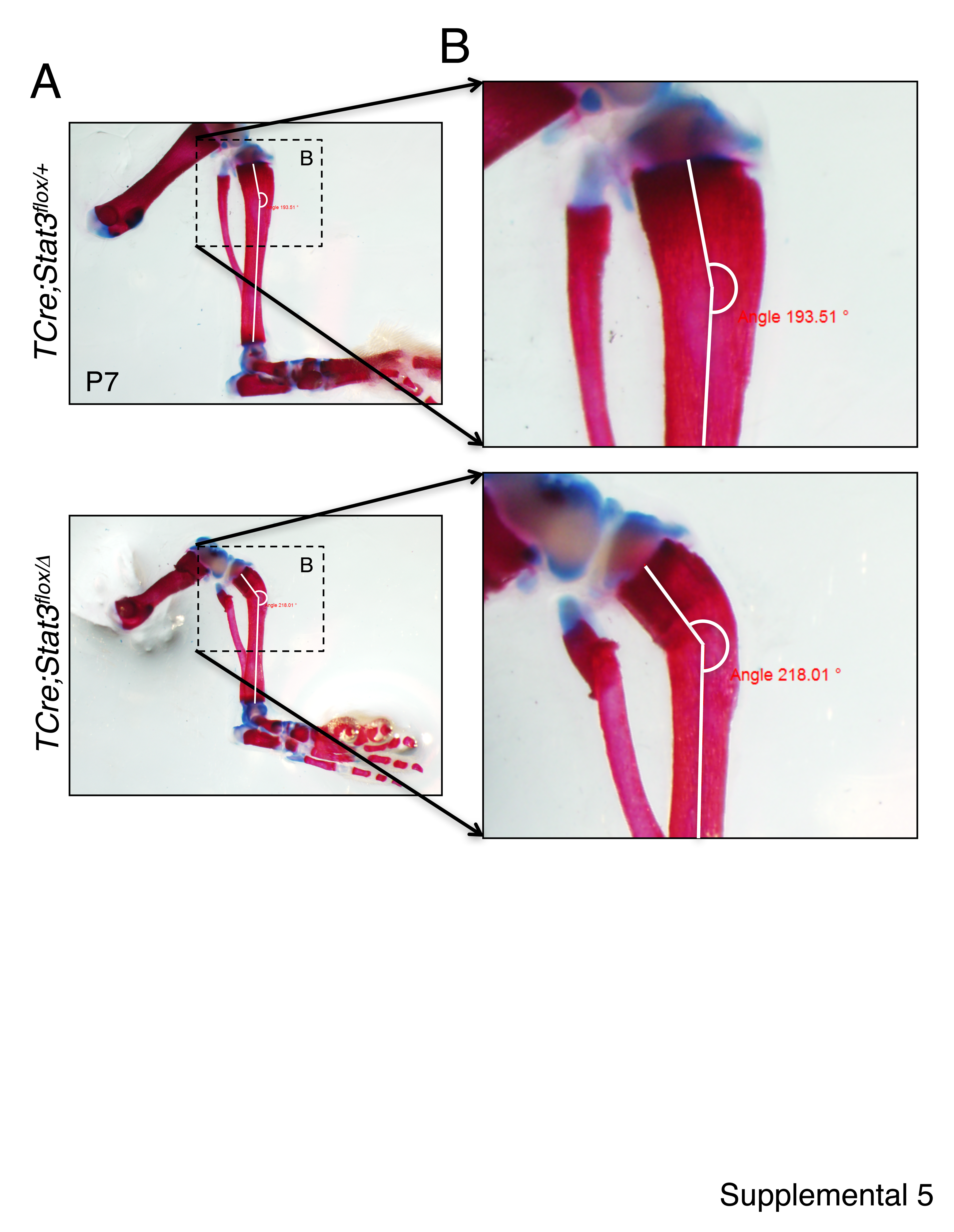

Supplement: S5 Fig — (A) Representative images and captured angular measurements of Alizarin Red/Alcian Blue-stained hindlimbs from P7 littermates. (B) Magnification of insets marked in A demonstrating representative points of measurement. (TIF) [file pgen.1006610.s005.tif]

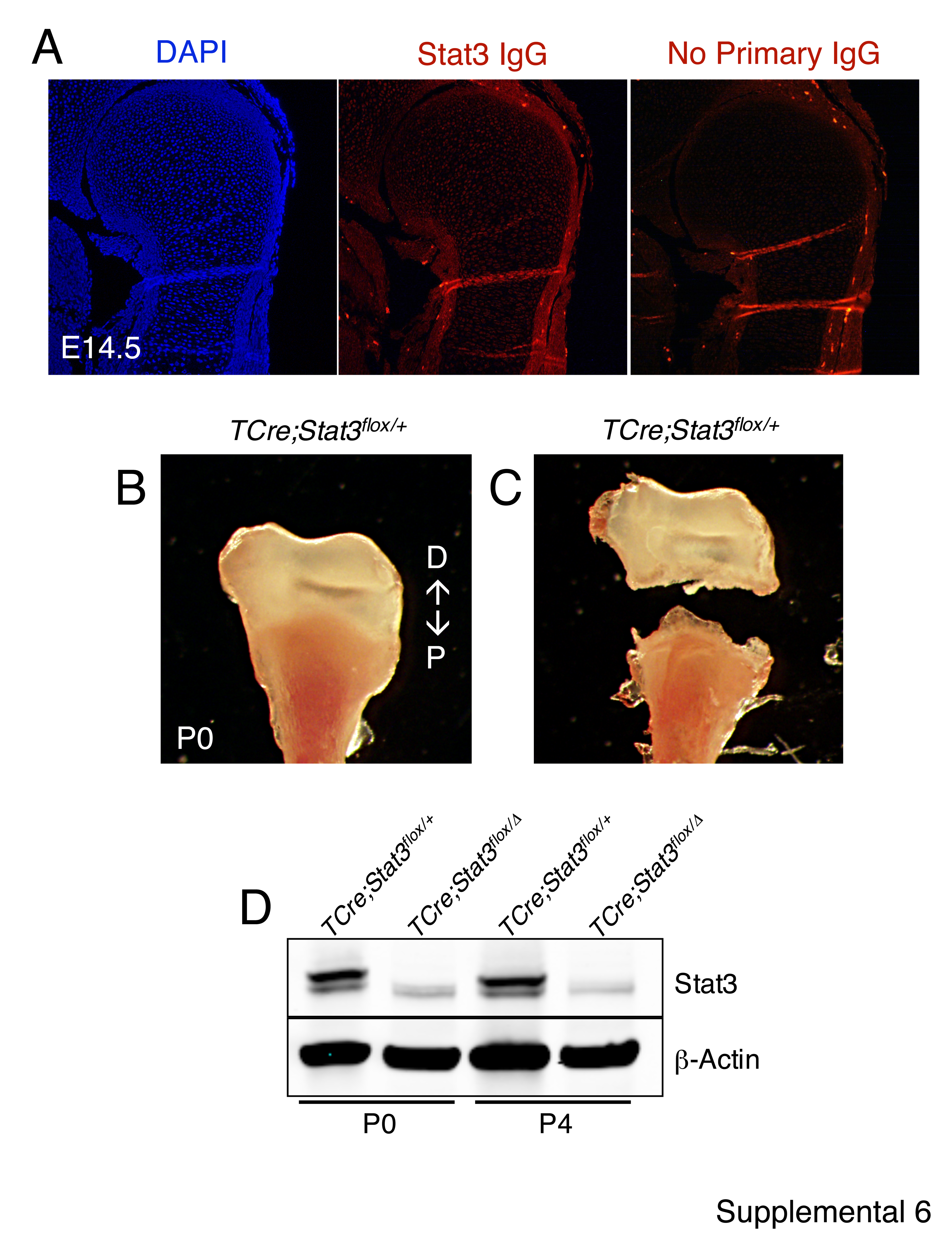

Supplement: S6 Fig — (A) Immunofluorescent analysis of Stat3 in serial sections from E14.5 control proximal humeri. (B and C) Representative depiction of distal humerus before and after epiphyseal dissection. (D) Representative immunoblot analysis of lysates generated from epiphyseal dissections from TCre;Stat3flox/+ TCre;Stat3flox/Δ noted in C, at indicated ages. (TIF) [file pgen.1006610.s006.tif]

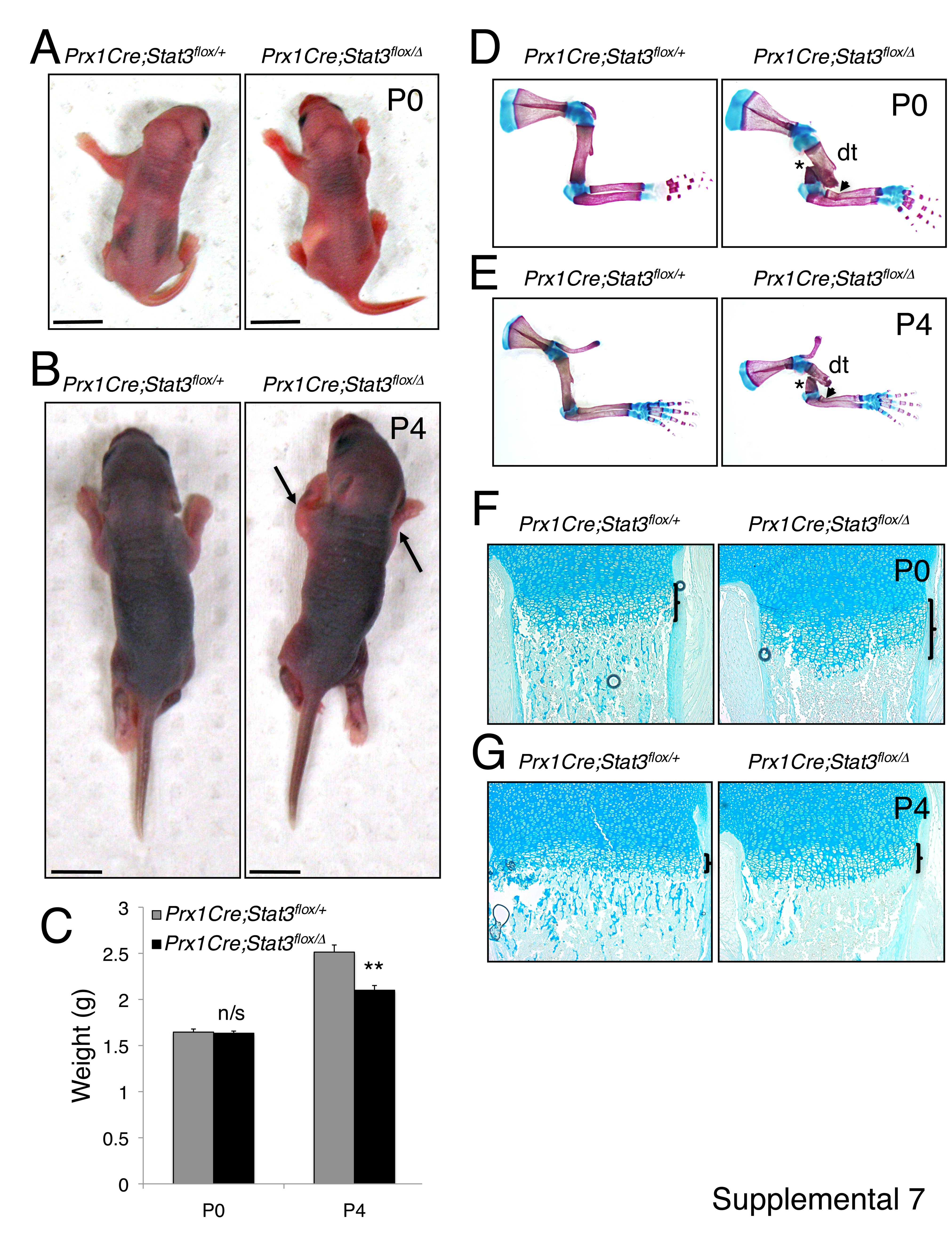

Supplement: S7 Fig — (A and B) Representative images of littermate matched control and Prx1Cre;Stat3flox/Δ mutant mice at P0 and P4. Arrows demonstrate bowing of forelimbs, bar = 1cm. (C) Chart depicting average weights of indicated genotypes in aging mice. Error bars are SEM, n/s—not significant, **p<0.01. (D and E) Alizarin Red/Alcian Blue-stained skeletal preps demonstrating bending and spontaneous fracture of forelimbs in Prx1Cre;Stat3flox/Δ mice at indicated ages. Arrows indicate fractures of radius/ulna, asterisk denotes humerus, dt—deltoid tuberosity. (F and G) Alcian Blue-stained longitudinal sections of the proximal humeri of control and mutant Prx1Cre;Stat3flox/Δ mice at indicated ages. Brackets denote the length of the hypertrophic chondrocyte region. (TIF) [file pgen.1006610.s007.tif]

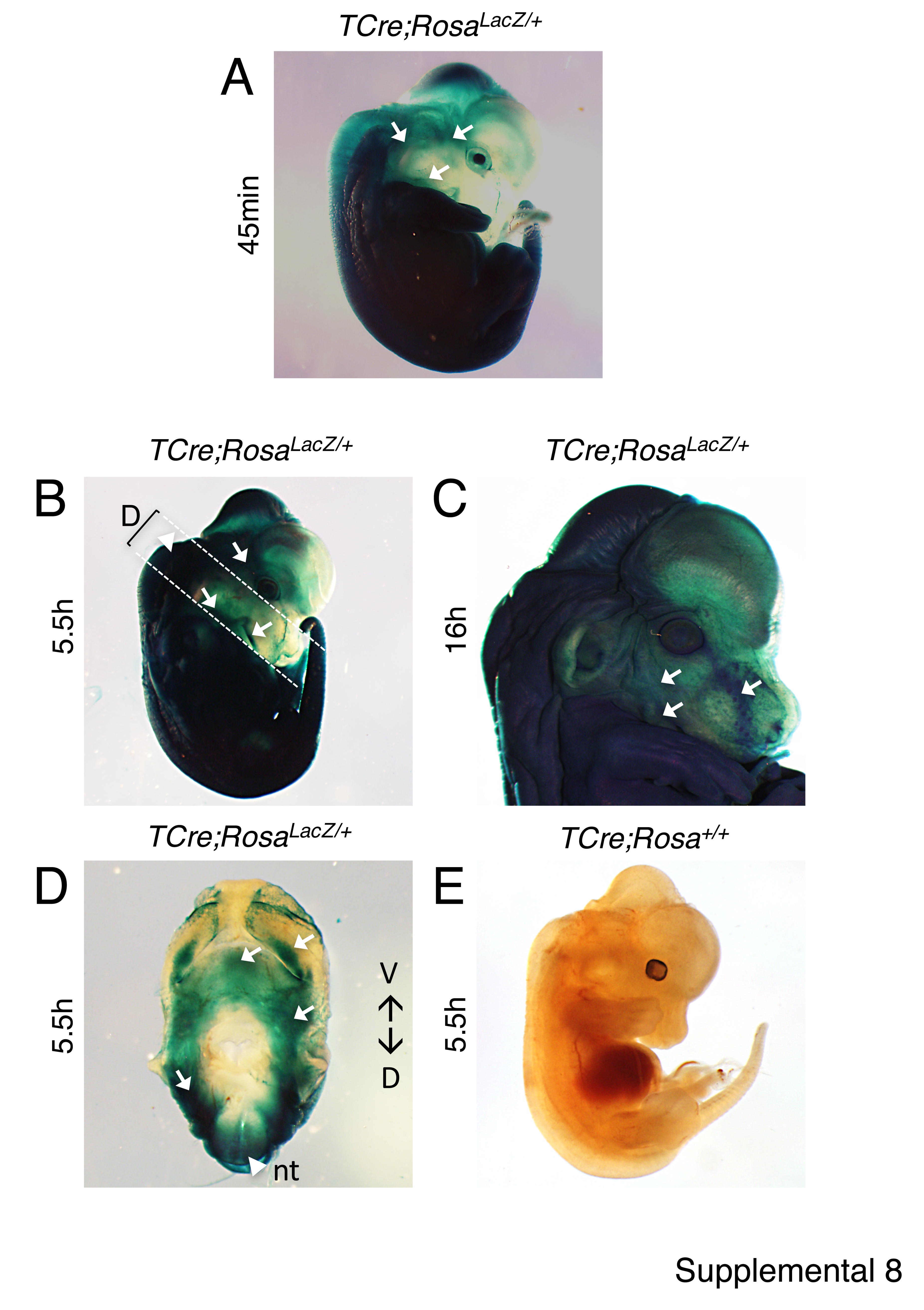

Supplement: S8 Fig — (A-C) Representative whole mount β-galactosidase staining of E12.5 embryo from TCre driver crossed with RosaLacZ carriers at indicated assay lengths. (D) Caudal aspect of transverse section from region denoted in panel B. (E) Representative negative control embryo demonstrating specificity of β-galactosidase protocol. Arrowheads indicate dorsal neural tube (nt) recombination. Arrows indicate likely cranial neural crest locations where TCre is active and overlap with observed reduction of Sox9 mRNA in Fig 6A. (TIF) [file pgen.1006610.s008.tif]

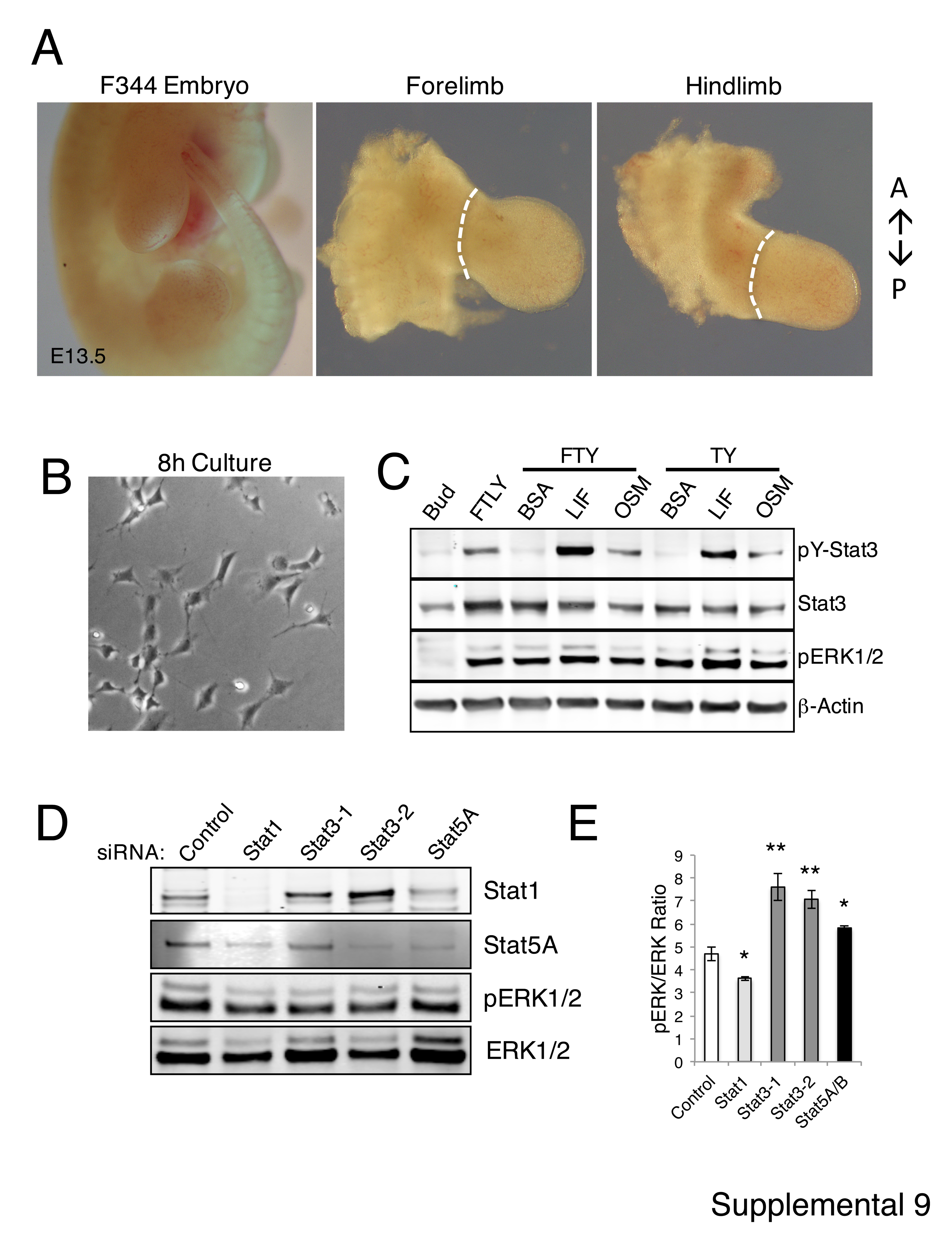

Supplement: S9 Fig — (A) Representative image of E13.5 F344 rat embryo and limb buds. Dotted lines indicate point of dissection for culture material. (B) Representative image of monolayer generated 8h post-dissection and culture. (C) Representative immunoblot demonstrating early induction of Stat3 activation in response to indicated stimuli in rat limb bud cells. F—Fgf2 (50ng/ml), T—TGFα (10ng/ml), Y—Y27632 (10μM). (D) Representative immunoblot demonstrating presence of indicated proteins in response to indicated siRNA treatments. (E) Quantitation of pERK1/2 levels in D. Error bars are SEM, *p<0.05, **p<0.01. (TIF) [file pgen.1006610.s009.tif]

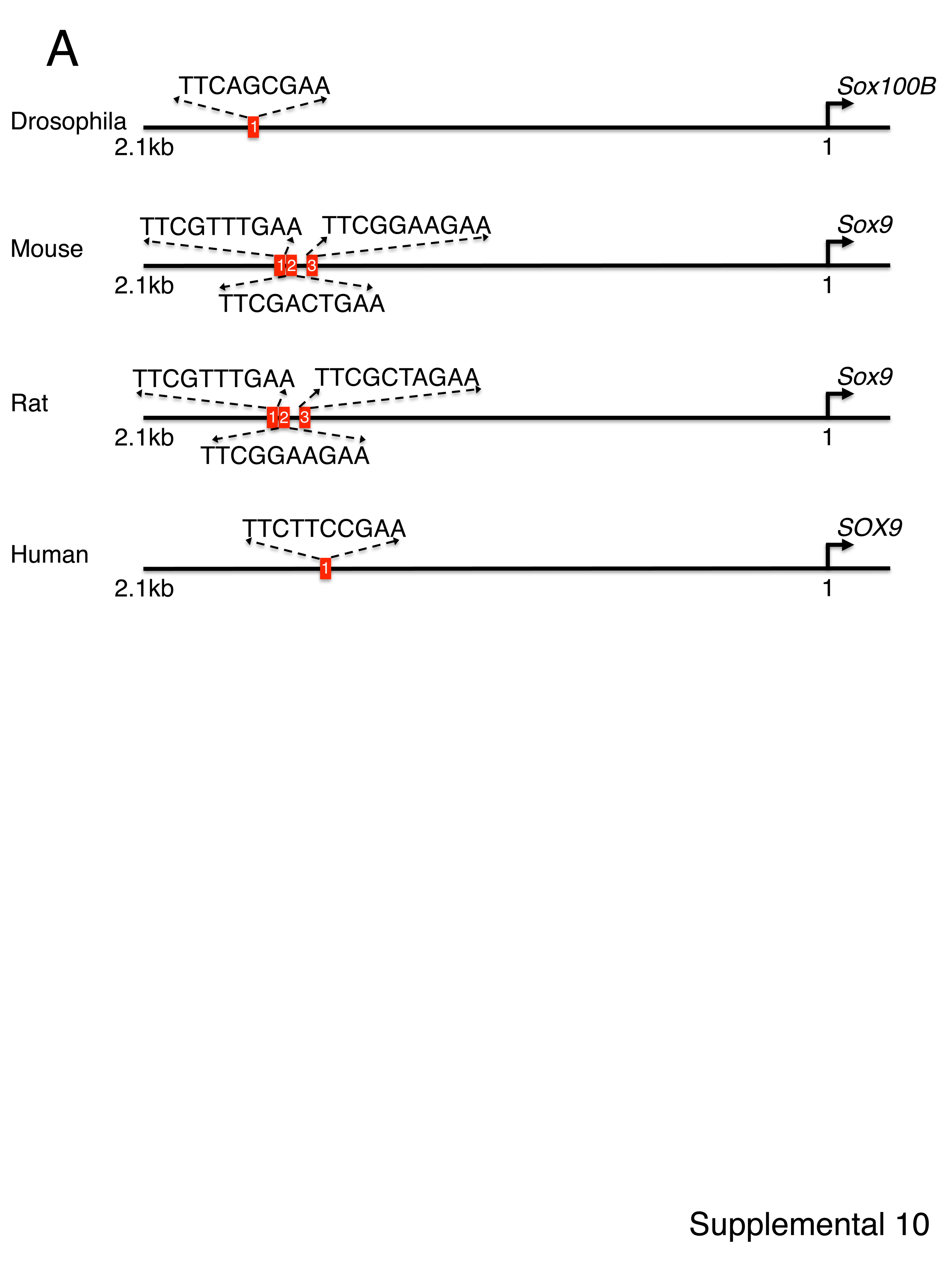

Supplement: S10 Fig — (A) Schematic of immediate upstream Sox9 homolog loci across indicated species. Red blocks indicate putative Stat DNA binding elements. (TIF) [file pgen.1006610.s010.tif]

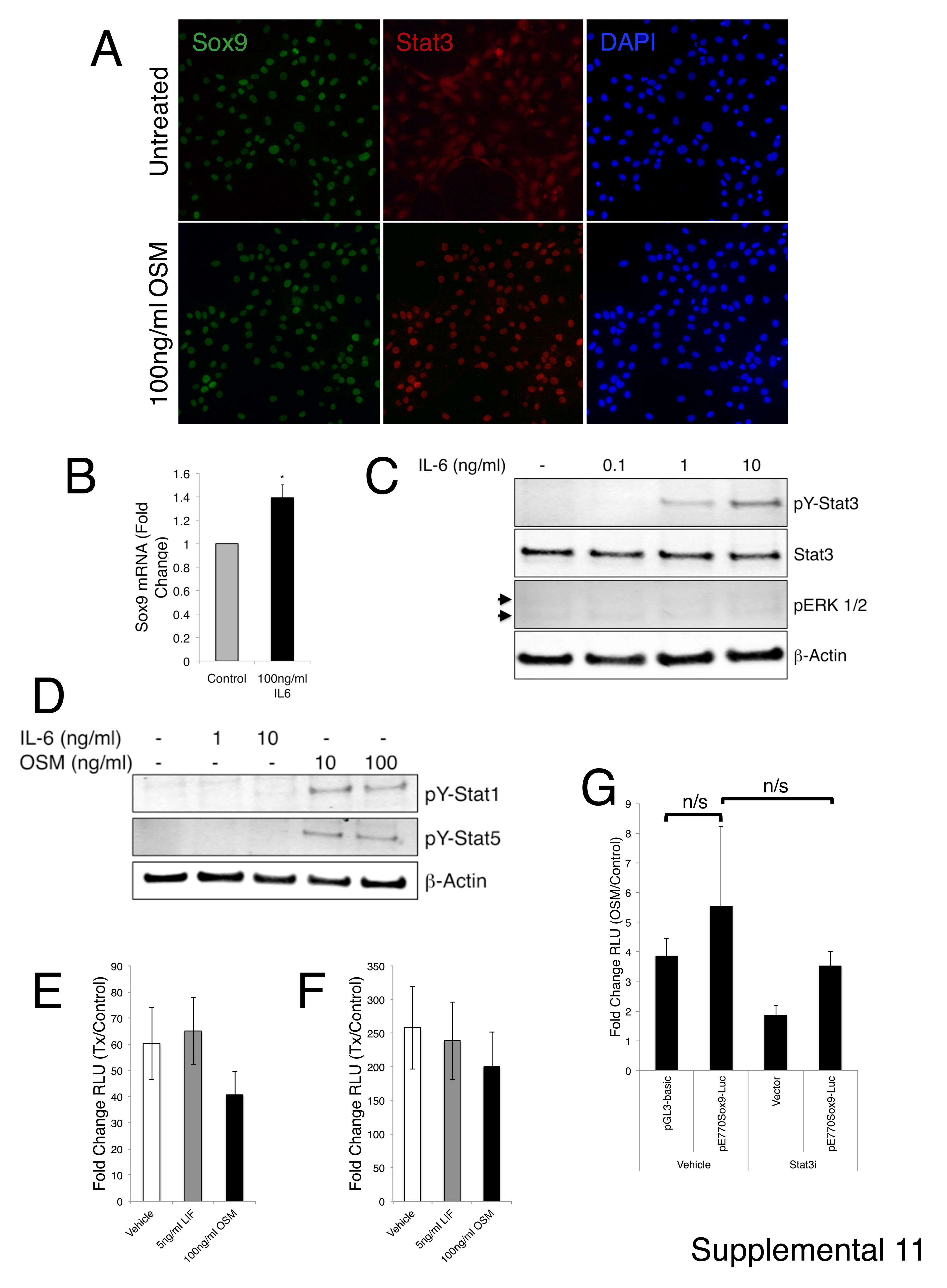

Supplement: S11 Fig — (A) Immunofluorescent analysis of Sox9 and Stat3 protein localization in NIH3T3 cells in response to stimulation by OSM for 30 minutes. (B) Total RNA isolated from control or IL-6 treated NIH3T3 cells analyzed for Sox9 expression by quantitative RT-PCR after 30 minutes. Error bars represent SEM, *p<0.05. (C and D) Immunoblot analysis depicting activation of indicated proteins in response to IL-6 or OSM treatment at specified doses. Arrowheads indicate doublet isoforms of ERK. (E and F) Analysis of a Sox9 promoter-driven luciferase construct in response to indicated treatment for 24h in rat limb bud cells grown in basal TY media (E) or FTLY media (F). Error bars are SEM. (G) Fold change analysis of a Sox9 promoter-driven luciferase construct lacking the Stat-binding regions in response to control or OSM treatment in the presence of a Stat3 inhibitor for 24h. Error bars are SEM, n/s—not significant. (TIF) [file pgen.1006610.s011.tif]

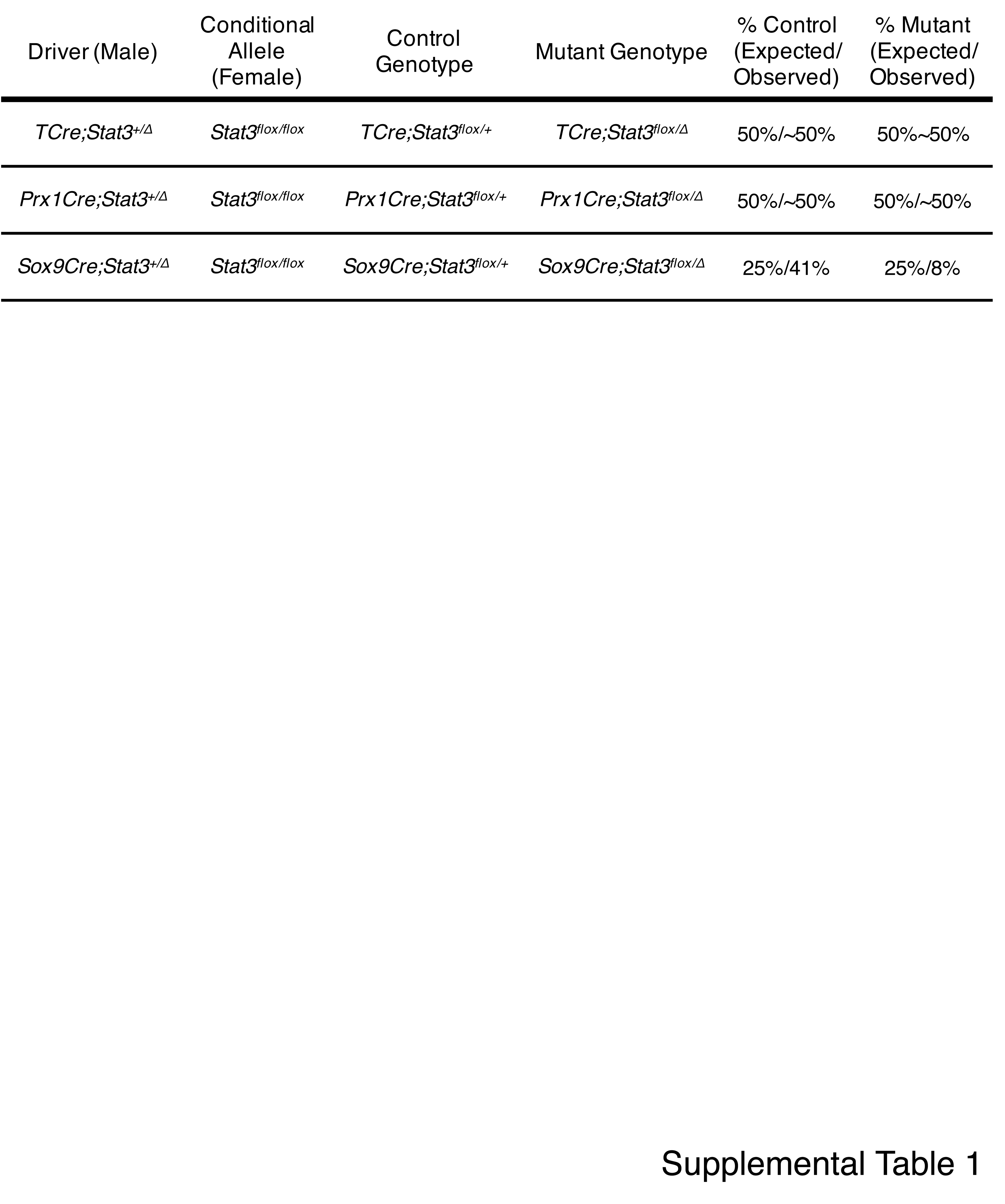

Supplement: S1 Table — TCre and Prx1Cre driver males were homozygous for the Cre transgene, where Sox9Cre males were heterozygous. Expected and observed offspring ratios at birth are indicated. (TIF) [file pgen.1006610.s012.tif]
